# The maternal–fetal interface is an early hematopoietic niche enriched in distinct erythro-myeloid progenitors

**DOI:** 10.1038/s41590-026-02683-4

**Published:** 2026-10-26

**Authors:** Rohit Jain, Shweta Tikoo, Carlos Márquez-Burgoa, Stephanie A. Gerhofer, Brendon Martinez, Mariagiovanna Barresi, Dörte Symmank, Minoo Ghannadan, Barbara Öhler, Anna-Lena Höbler, Rafael de Freitas e Silva, Dirk Baumjohann, Susanne Feil, Robert Feil, Sylvia Knapp, Philipp Starkl, Wilfried Ellmeier, Steffen Jung, Jürgen Pollheimer, Stuart T. Fraser, Wolfgang Weninger

**Affiliations:** 1 Department of Dermatology, Medical University of Vienna, Vienna, Austria; 2 Centenary Institute, Faculty of Medicine and Health, University of Sydney, Camperdown, New South Wales Australia; 3 Sydney Medical School, University of Sydney, Camperdown, New South Wales Australia; 4 Interfakultäres Institut für Biochemie (IFIB), Universität Tübingen, Tübingen, Germany; 5 Department of Obstetrics and Gynecology, Reproductive Biology Unit, Maternal–Fetal Immunology Group, Medical University of Vienna, Vienna, Austria; 6 Center for Pathophysiology, Infectiology and Immunology, Institute of Immunology, Medical University of Vienna, Vienna, Austria; 7 Medical Clinic III for Oncology, Hematology, Immuno-Oncology and Rheumatology, University Hospital Bonn, University of Bonn, Bonn, Germany; 8 Research Division of Infection Biology, Department of Medicine I, Medical University of Vienna, Vienna, Austria; 9 Department of Immunology and Regenerative Biology, Weizmann Institute of Science, Rehovot, Israel; 10 School of Medical Sciences, University of Sydney, Sydney, New South Wales Australia; 11 University of Sydney Nano Institute, University of Sydney, Sydney, New South Wales Australia

**Keywords:** Haematopoiesis, Innate immunity

## Abstract

Embryonic hematopoiesis is thought to occur exclusively in the extraembryonic yolk sac before hematopoietic stem cells (HSCs) arise in the aorta–gonad–mesonephros region. Here we identify a previously unrecognized hematopoietic site at the maternal–fetal interface in mice, termed MIXER (Maternally Interfaced eXtraEmbRyonic) tissue, which develops contemporaneously with the yolk sac and exhibits robust hematopoietic activity. MIXER contains abundant hematopoietic precursors, including classical CSF1R^+^ erythro-myeloid progenitors and a unique embryonic CD4^+^CSF1R^−^ Erythro-Myeloid PRogenitOR (eEMPROR) population. Lineage-tracing experiments demonstrate that eEMPROR cells contribute to select macrophage subpopulations during fetal development, primarily in the perivascular space. Notably, early human placental villi, before HSC emergence, also harbor CD4^+^ nucleated erythrocytes, indicating a conserved hematopoietic role for the maternal–fetal interface. These findings establish MIXER as an anatomically distinct niche for embryonic hematopoiesis and expand the developmental map of blood cell emergence in mammals.

## Abstract (web summary)

Jain et al. identify a unique extraembryonic hematopoietic niche at the maternal–fetal interface that is distinct from the yolk sac and is capable of producing CD4^+^CSF1R^−^ erythro-myeloid progenitors.

## Main

The hematopoietic system is a highly organized and tightly regulated network responsible for the lifelong production of blood cells^1,2^. It generates innate and adaptive immune cells essential for host defense^3^ as well as red blood cells (RBCs) that deliver oxygen throughout the body^3^. More than a hundred billion blood cells are produced daily in humans^4,5^, underscoring the central role of hematopoiesis within specialized microenvironments^6^. In adults, the bone marrow serves as the predominant hematopoietic organ^7,8^. During embryogenesis, however, the absence of a stable hematopoietic site presents a developmental challenge, made more complex by the increasing demands for oxygenation^9^ and phagocytic clearance during tissue remodeling. Consequently, hematopoietic activity transitions across several extraembryonic and embryonic tissues in a tightly timed sequence^10^.

The extraembryonic yolk sac is widely considered the earliest and initially the sole site of embryonic hematopoiesis. It produces primitive erythrocytes and the first erythro-myeloid progenitors (EMPs), independently of hematopoietic stem cells (HSCs)^11,12^. Primitive hematopoiesis begins at embryonic day 7.25 (E7.25) to E7.5, generating primitive erythrocytes and primitive macrophage progenitors^13^, followed by EMPs (hereafter CSF1R^+^ EMPs) at E8.5. These CSF1R^+^ EMPs give rise to long-lived tissue-resident macrophages, including microglia, Langerhans cells, alveolar macrophages and Kupffer cells^14,15^, through developmental pathways distinct from those of HSC-derived lineages^14,16,17^. Many yolk sac-derived macrophages, such as microglia, persist throughout life without adult replacement^14,15,18–20^. Subsequently, hematopoietic activity has been detected in additional sites, including the allantois^21,22^, aorta–gonad–mesonephros (AGM) region^23–26^ and placenta^27–35^, with the AGM generating the first HSCs that seed the fetal liver^13^. A few days before birth, HSCs relocate to the bone marrow, establishing the lifelong hematopoietic niche.

Despite the transient nature of hematopoiesis within each embryonic tissue, the progenitors they produce have an enduring influence, contributing to lifelong tissue homeostasis and influencing disease susceptibility^2^. For example, microglia derived from CSF1R^+^ EMPs self-maintain in the brain, and their dysregulation is implicated in Alzheimer’s disease^36^ and other neuroinflammatory disorders^37^. Similarly, AGM-derived HSCs support fetal liver hematopoiesis and ultimately populate adult bone marrow niches^38,39^. Thus, embryonic hematopoiesis, although spatially and temporally dynamic, forms the foundation for postnatal immunity and homeostasis, and its disruption is linked to age-related pathologies. Despite its importance, the prevailing model assumes that all early hematopoietic activity originates in the yolk sac, leaving a substantial gap in our understanding of how the embryo meets its rapidly increasing demand for erythroid and myeloid cells before HSCs emerge.

Here, we identify a previously unrecognized site of embryonic hematopoiesis. This region, which is anatomically distinct from the yolk sac, is termed MIXER (Maternally Interfaced eXtraEmbRyonic) tissue and encompasses the extraembryonic compartment located at the maternal–fetal interface. We show that MIXER supports the development of hematopoietic progenitor cells. It is spatially separate from but temporally concurrent with the yolk sac, establishing it as one of the earliest hematopoietic sites. MIXER is enriched in multiple erythroid and myeloid progenitors and represents an entirely unexplored niche within early ontogeny. In addition to conventional CSF1R^+^ EMPs, MIXER uniquely contains embryonic CD4^+^CSF1R^−^ EMPs, termed eEMPROR cells, which are absent from the yolk sac. The identification of MIXER as a new hematopoietic compartment has broad implications, updating fundamental models of early blood development and introducing a new extraembryonic source of progenitors that may reshape our understanding of developmental biology and inform future clinical applications.

## Results

### Identification of MIXER tissue

**Fig. 1:**
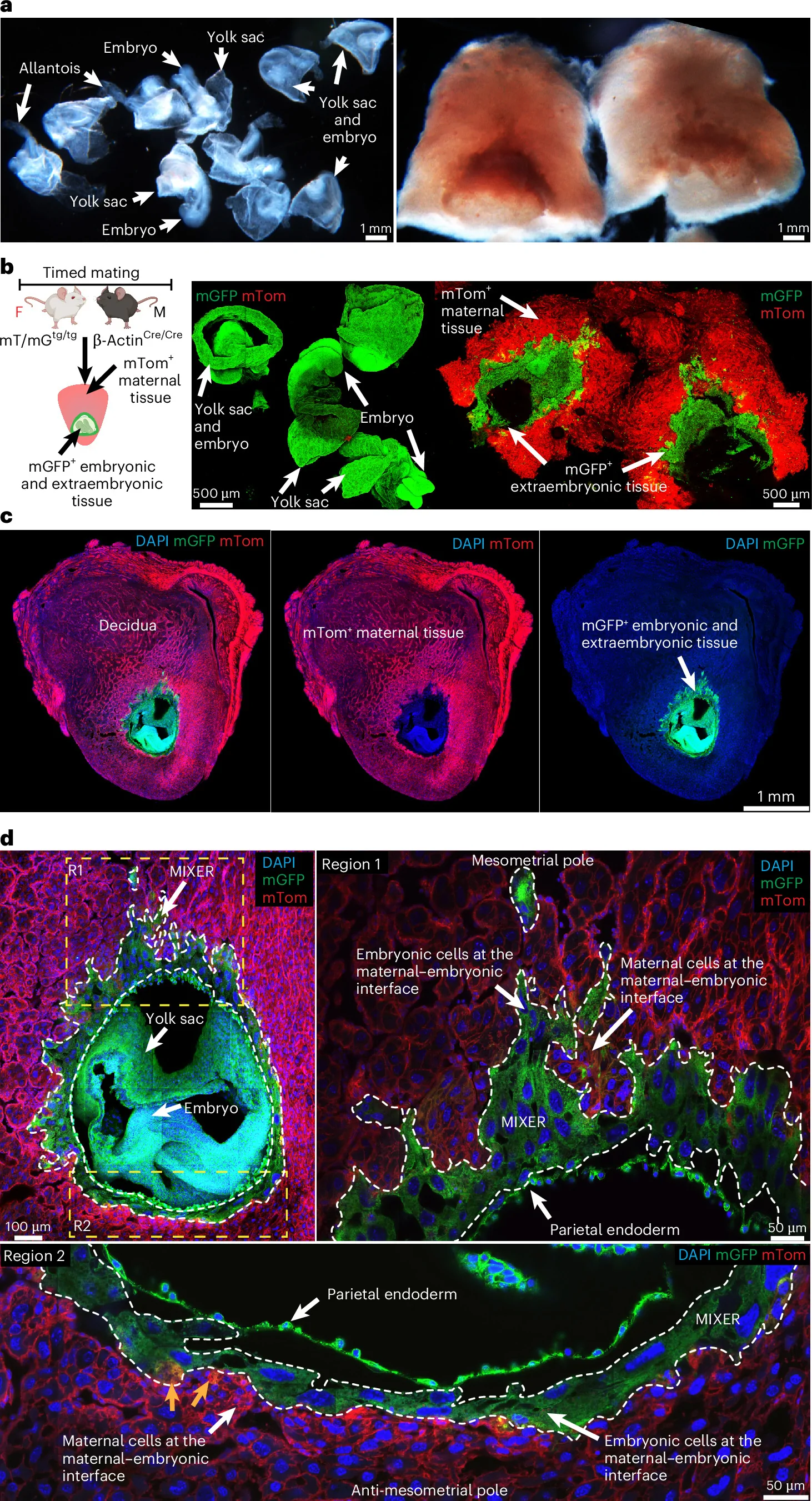
High-resolution imaging of the maternal–fetal interface. **a**, Photomicrographs of microdissected mouse conceptuses at 8.5 days post coitus (E8.5). Left, image showing the intact embryo and yolk sacs collected from several conceptuses. Right, image showing the vascular bed (red) within one of the conceptuses. **b**, Left, schematic representation of the timed mating strategy allowing for the identification of both maternal (mTom^+^) and embryonic (mGFP^+^) cells in E8.5 β-actin-Cre:mT/mG embryos. M, male; F, female. Middle, confocal image highlighting the minimal mTom^+^ tissue (maternal) associated with the microdissected embryo and yolk sac tissue (mGFP^+^). Right, image showing that a large proportion of extraembryonic tissue (mGFP^+^) can be observed enmeshed within the maternal uterine tissue (mTom^+^). **c**, Tiled confocal images highlighting mTom and mGFP expression within cells of maternal and embryonic origin, respectively. **d**, Higher-resolution images of the embryonic and MIXER tissue (region between the white dotted lines) within the decidua. Region 1 shows higher-magnification images of MIXER at the mesometrial pole, highlighting the infiltrating embryonic cells within the maternal tissue, while a distinct boundary is observed at the anti-mesometrial pole (region 2). Orange arrows indicate the presence of occasional cells with both mGFP^+^ and mTom^+^ membranes at the anti-mesometrial pole. In **a** and **b**, the images are representative of two independent timed matings. In **c** and **d**, the images are representative of three independent conceptuses from three independent timed matings. Icons in **b** created in BioRender; Jain, R. https://biorender.com/f6aluj6 (2026).

During early ontogeny, hematopoietic precursors are thought to arise exclusively within the extraembryonic yolk sac^14,17,40^. In our accompanying study, we identified a population of EMP-like cells (EMPROR) that arises from the CD4 lineage in adult bone marrow^41^. To investigate whether analogous progenitors might also exist during early development, we collected E8.5 embryos with their yolk sacs intact (Fig. 1a, left), minimizing disruption to the surrounding maternal uterine tissue for detailed analysis (Fig. 1a, right). Macroscopic examination consistently revealed a markedly vascularized region at the site of embryonic implantation, visible as a dense red zone (Fig. 1a, right). It is well established that the implantation stroma contains rich maternal vasculature, immune cells and embryo-derived trophoblasts; however, we wondered whether this region might also harbor hematopoietic cells of embryonic origin (analogous to the yolk sac) and thereby contribute to early hematopoiesis.

**Extended Data Fig. 1:**
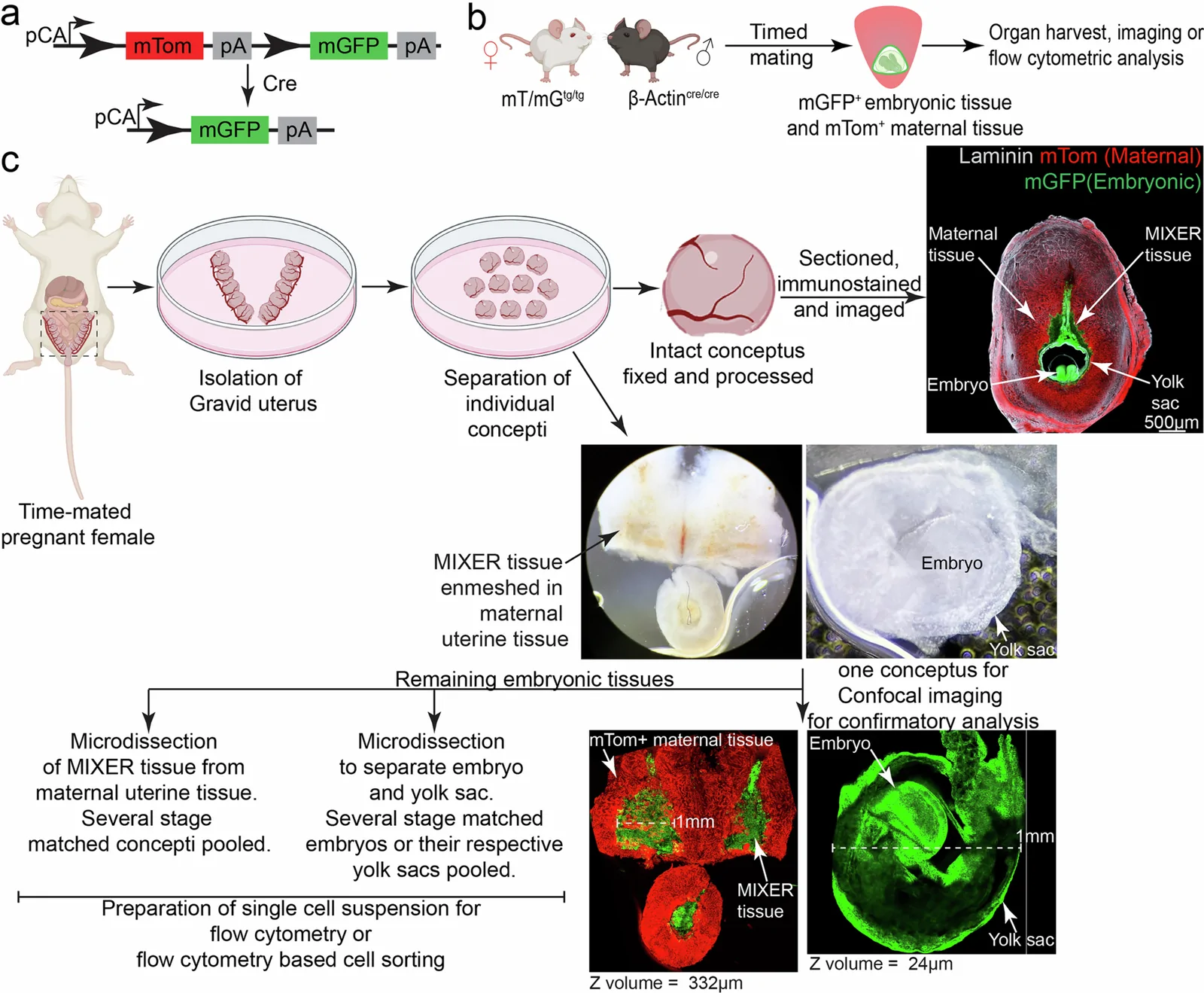
Embryo microdissection. **a**, Schematic representation of the mT/mG construct before and after Cre-mediated recombination; **b** and **c**, Schematic diagram highlighting the methodology used for timed mating and tissue harvest for either immunostaining and confocal microscopy or flow cytometry. Panel **a** adapted with permission from ref. ^43^, Wiley; icons in **a**–**c** created in BioRender; Jain, R. https://biorender.com/f6aluj6 (2026).

The detailed characterization of cellular lineages at the implantation site has historically been limited by the difficulty of unequivocally distinguishing embryonic cells from maternal cells. To overcome this challenge, we paired β-actin-Cre male mice with mT/mG female reporter mice for timed mating (Fig. 1b, left, and Extended Data Fig. 1). β-Actin-Cre transgenic mice exhibit ubiquitous Cre activity from the blastocyst stage^42^, whereas the mT/mG double reporter mouse strain shows global expression of membrane-targeted tandem dimer (td)Tomato (mTom) before, and membrane-targeted EGFP (mGFP) expression after, Cre-mediated excision^43^. Cre excises the floxed mTom cassette, switching expression to mGFP (Extended Data Fig. 1a). Consequently, embryos from these crosses express mGFP in embryonic tissues and mTom in maternal tissues, enabling precise discrimination between the two compartments throughout development (Fig. 1b,c).

Using this strategy, we sectioned the whole gravid uterus and performed confocal laser scanning microscopy (Fig. 1c,d). Indeed, double-transgenic embryos displayed a clear separation between mTom^+^ maternal cells and mGFP^+^ embryonic cells (Fig. 1c). At E8.5, we observed that the embryonic cells outline the uterine lumen in its entirety^44^ (Fig. 1c,d and Extended Data Fig. 1), with notable site-specific differences. At the implantation site (that is, the mesometrial pole, the site of placental development), embryonic cells intermingled with maternal cells (Fig. 1d, region 1), indicating their active invasion into the uterine wall. Surprisingly, however, at sites more distant from implantation (that is, the anti-mesometrial pole), embryonic and maternal cells segregated into separate layers; occasional double labeling of cell membranes was observed in juxtaposed cells at the border (Fig. 1d, region 2, orange arrows). For simplicity, we refer to this tissue as MIXER (Fig. 1d). Notably, we did not observe the presence of mTom^+^ maternal cells within the MIXER tissue.

### MIXER tissue harbors embryo-derived hematopoietic cells

**Fig. 2:**
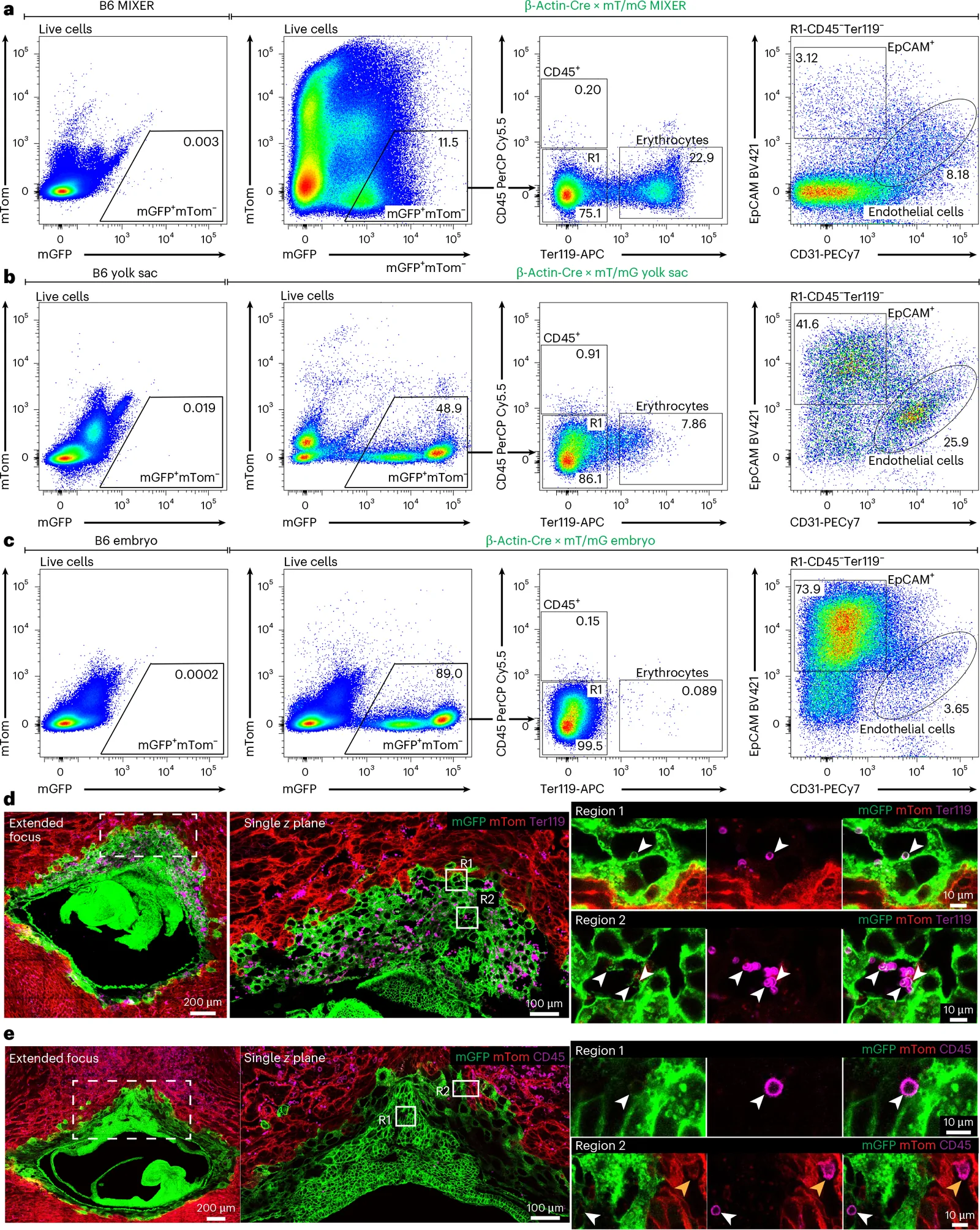
Characterization of the MIXER tissue. **a**–**c**, Flow cytometric analysis of MIXER (**a**), yolk sac (**b**) and embryo proper (**c**) tissues from E8.5 C57BL/6JRj (B6, control) or β-actin-Cre:mT/mG embryos. The flow plots highlight the expression of various hematopoietic (CD45 and Ter119) and stromal (CD31 and EpCAM) markers within the MIXER, yolk sac and embryonic tissues. Data are representative of six to nine pooled embryos per litter from three independent experiments. E8.5 embryos were staged with six to eight somite pairs. **d**,**e**, Confocal images of immunostained sections of the conceptus, highlighting the presence of embryonic (mGFP^+^mTom^−^) Ter119^+^ erythrocytes (**d**) and CD45^+^ leukocytes (**e**) within MIXER. White arrowheads indicate labeled embryonic cells, and orange arrowheads mark labeled maternal cells. In **d** and **e**, the images are representative of five different embryos from two independent timed matings. Insets show a higher magnification of a few mGFP^+^ cells, highlighting the expression of the indicated markers.

We next set out to investigate the nature of embryo-derived cells within the MIXER tissue compared to those in the yolk sac. To achieve this, we microdissected embryonic tissues (Extended Data Fig. 1) from β-actin-Cre × mT/mG timed matings, as well as C57BL/6JRj embryos as controls, and performed flow cytometric analysis. These studies clearly indicated the presence of embryonic erythrocytes (mTom^−^ mGFP^+^CD45^−^Ter119^+^), leukocytes (mTom^−^ mGFP^+^CD45^+^), endothelial cells (mTom^−^ mGFP^+^CD45^−^Ter119^−^CD31^+^) and epithelial cells (mTom^−^ mGFP^+^CD45^−^Ter119^−^EpCAM^+^) in MIXER (Fig. 2a). The yolk sac comprised endothelial cells and a small population of EpCAM^+^ cells, CD45^+^ cells and Ter119^+^ erythrocytes (Fig. 2b). In contrast, the embryo proper harbored a robust EpCAM^+^ population, a small endothelial cell population and only very few erythrocytes (Fig. 2c). These data are consistent with the absence of fully functional vascular connections between the embryo and its extraembryonic tissues at this stage.

**Extended Data Fig. 2:**
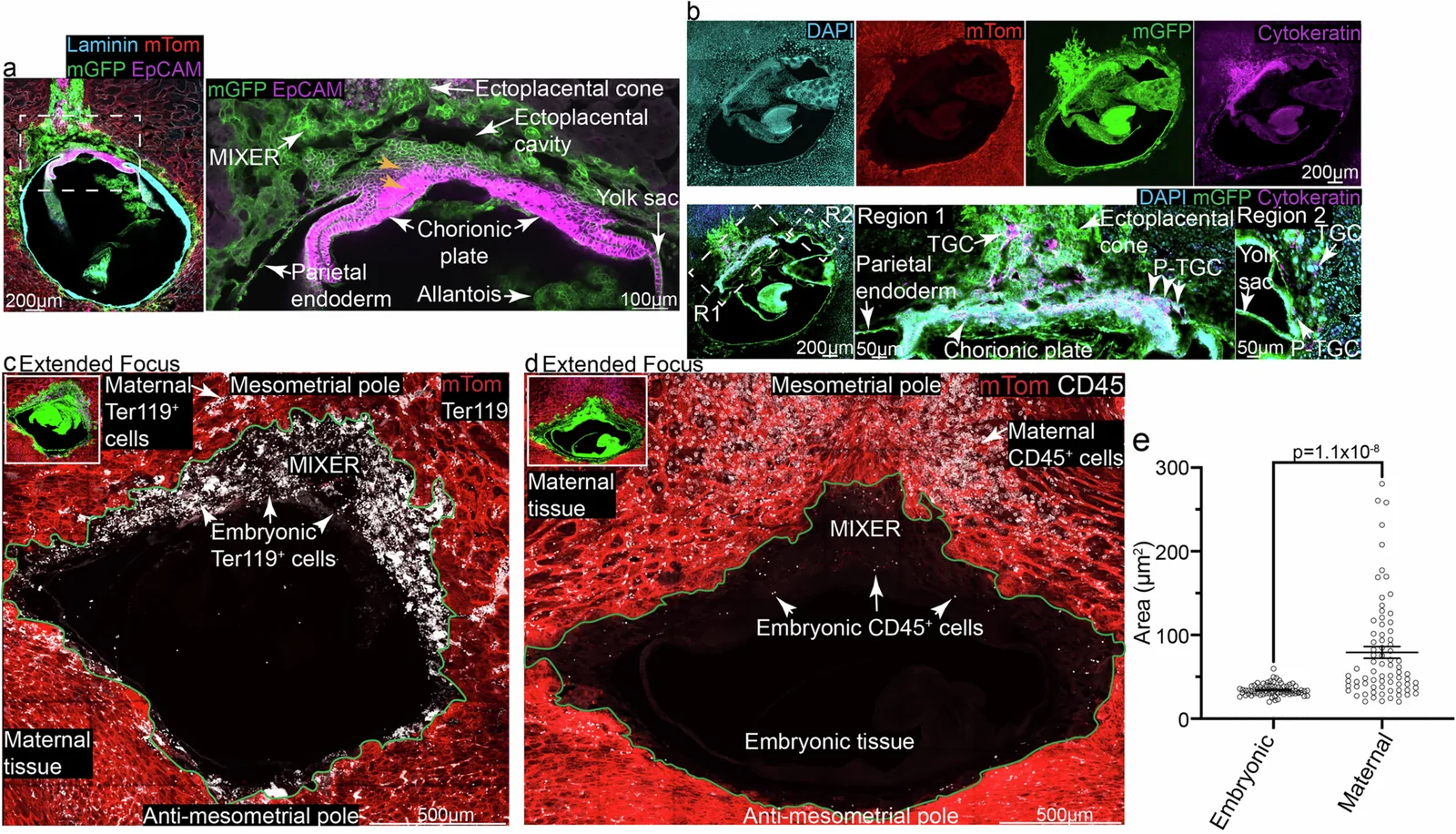
Characterization of MIXER tissue. **a** and **b**, Confocal imaging of E8.5 conceptus from β-actin-Cre x mT/mG timed matings, highlighting the presence of embryonic (mGFP^+^mTom^−^) EpCAM^+^ (Panel **a**) and pan-Cytokeratin^+^ (Panel **b**) cells within the MIXER tissue. TGC- Trophoblast giant cells; P-TGC- Parietal trophoblast giant cells. Images are representative of 4 independent embryos from 4 independent timed matings; **c** and **d**, Confocal image of E8.5 conceptus from β-actin-Cre x mT/mG timed matings, highlighting the localization of Ter119^+^ (Panel **c**) and CD45^+^ (Panel **d**) within the MIXER (region demarcated) and maternal tissue (mTom^+^). Data representative of 5 different embryos from 2 independent timed matings. The images do not show mGFP to highlight the numerous cells present within the MIXER tissue. The embryonic and extra-embryonic regions expressing mGFP can be observed within the insets. **e**, Cell area analysis between embryonic and maternal CD45^+^ cells. 75 cells per group were randomly selected from both tissues. Statistical significance was determined using two-tailed unpaired t-test with Welch’s correction and 95% confidence interval. Data are presented as mean ± standard error of the mean, with the symbols representing individual cells. Source data

Immunostaining of tissue sections for structural components at E8.5 highlighted the tissue-wide localization of epithelial cells (Extended Data Fig. 2a) and trophoblasts (Extended Data Fig. 2b) within MIXER. Both the yolk sac and the chorionic plate demonstrated high expression of EpCAM (Extended Data Fig. 2a). Consistent with previous reports^45,46^, we also observed the presence of pan-cytokeratin-positive parietal trophoblast giant cells at the interface of MIXER and the yolk sac (Extended Data Fig. 2b).

To provide spatial context for the positioning of erythrocytes and leukocytes within MIXER, we performed immunostaining of gravid uterine sections followed by high-resolution confocal microscopy. This highlighted the presence of numerous embryonic Ter119^+^ erythrocytes (Fig. 2d and Extended Data Fig. 2c) along with CD45^+^ leukocytes (Fig. 2e and Extended Data Fig. 2d). We observed that the Ter119^+^ erythrocytes and CD45^+^ leukocytes were evenly dispersed throughout the MIXER tissue rather than being confined to a specific subanatomical location (Extended Data Fig. 2c,d). These embryonic hematopoietic cells exhibited morphologies and densities distinct from those of their maternal counterparts (Fig. 2d,e and Extended Data Fig. 2c,d). For example, CD45^+^ leukocytes within the MIXER tissue were smaller in size than their maternal counterparts (Fig. 2e and Extended Data Fig. 2d,e).

These observations raise the possibility that MIXER is not merely a reservoir of blood cells but also a unique hematopoietic niche, prompting us to test its progenitor activity directly.

### Detection of hematopoietic activity within the MIXER tissue

**Fig. 3:**
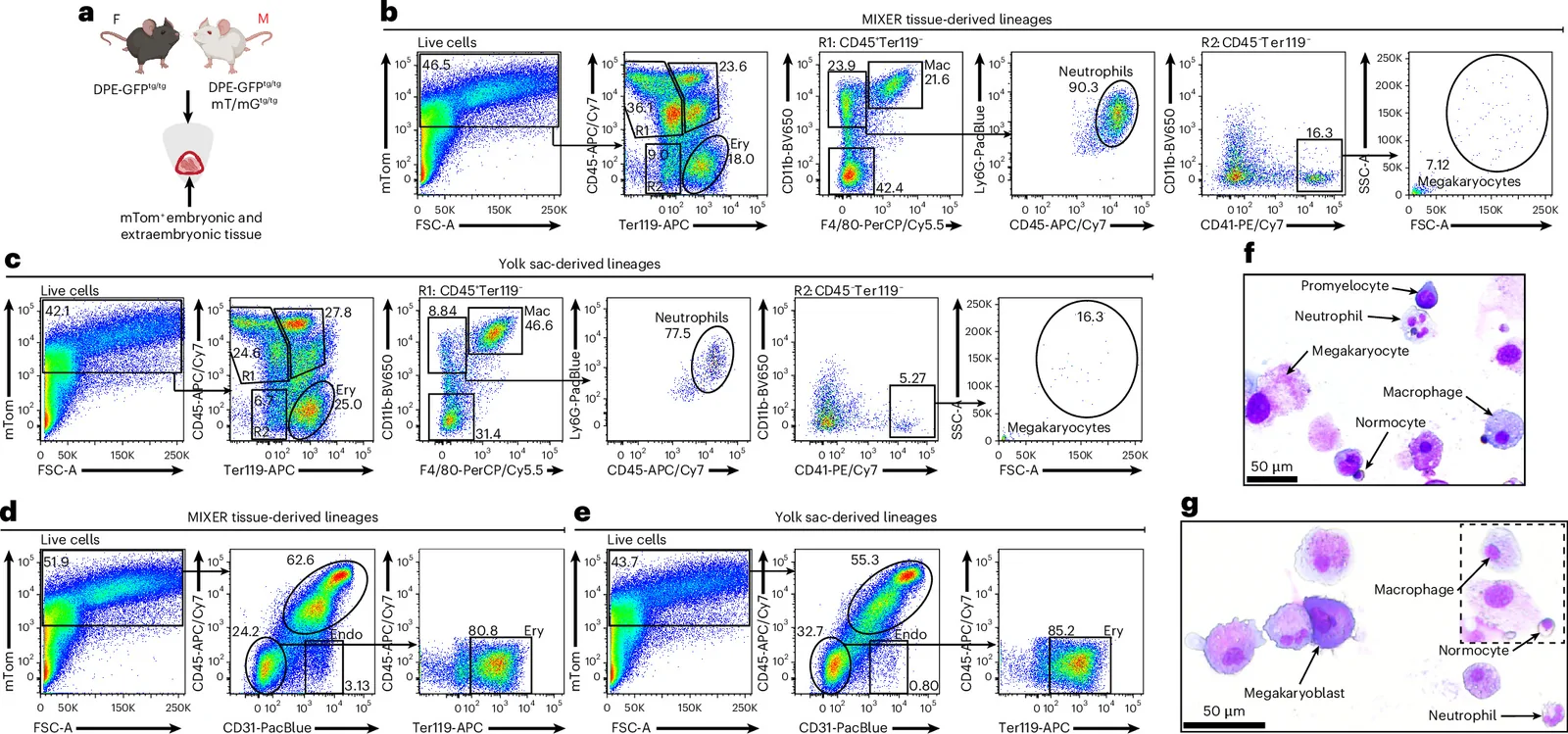
MIXER-derived cells exhibit hematopoietic potential. **a**, Schematic representation of the timed mating strategy in which a DPE-GFP^tg^^/^^tg^ female mouse is paired with a DPE-GFP^tg^^/^^tg^:mT/mG^tg^^/^^tg^ male mouse for timed mating. The strategy allows for robust identification of embryonic tissue (mTom^+^). **b**,**c**, Flow cytometric analysis of lineages generated after the culture of mTom^+^ cells sorted from MIXER (**b**) and the yolk sac (**c**). The sorted cells were cultured on an OP9 stromal layer in the presence of SCF, IL-3, IL-6, EPO, TPO, GM-CSF and M-CSF. Mac, macrophages; Ery, erythrocytes. **d**,**e**, Flow cytometric analysis of cell lineages arising from sorted mTom^+^ MIXER-derived cells (**d**) and yolk sac-derived cells (**e**). The coexpression of CD45 and CD31 in **d** and **e** suggests the potential recent emergence of these cells from the hemogenic endothelium. Endo refers to CD45^−^CD31^+^ endothelial cells. **f**,**g**, Giemsa staining of colonies depicting erythroid, megakaryocytic and myeloid lineage potential, generated after the culture of mTom^+^ cells sorted from MIXER (**f**) and yolk sac (**g**) tissues. **b**–**g**, Representative data from one of three independent sorting and culture experiments are shown. Icons in **a** created in BioRender; Jain, R. https://biorender.com/f6aluj6 (2026).

**Extended Data Fig. 3:**
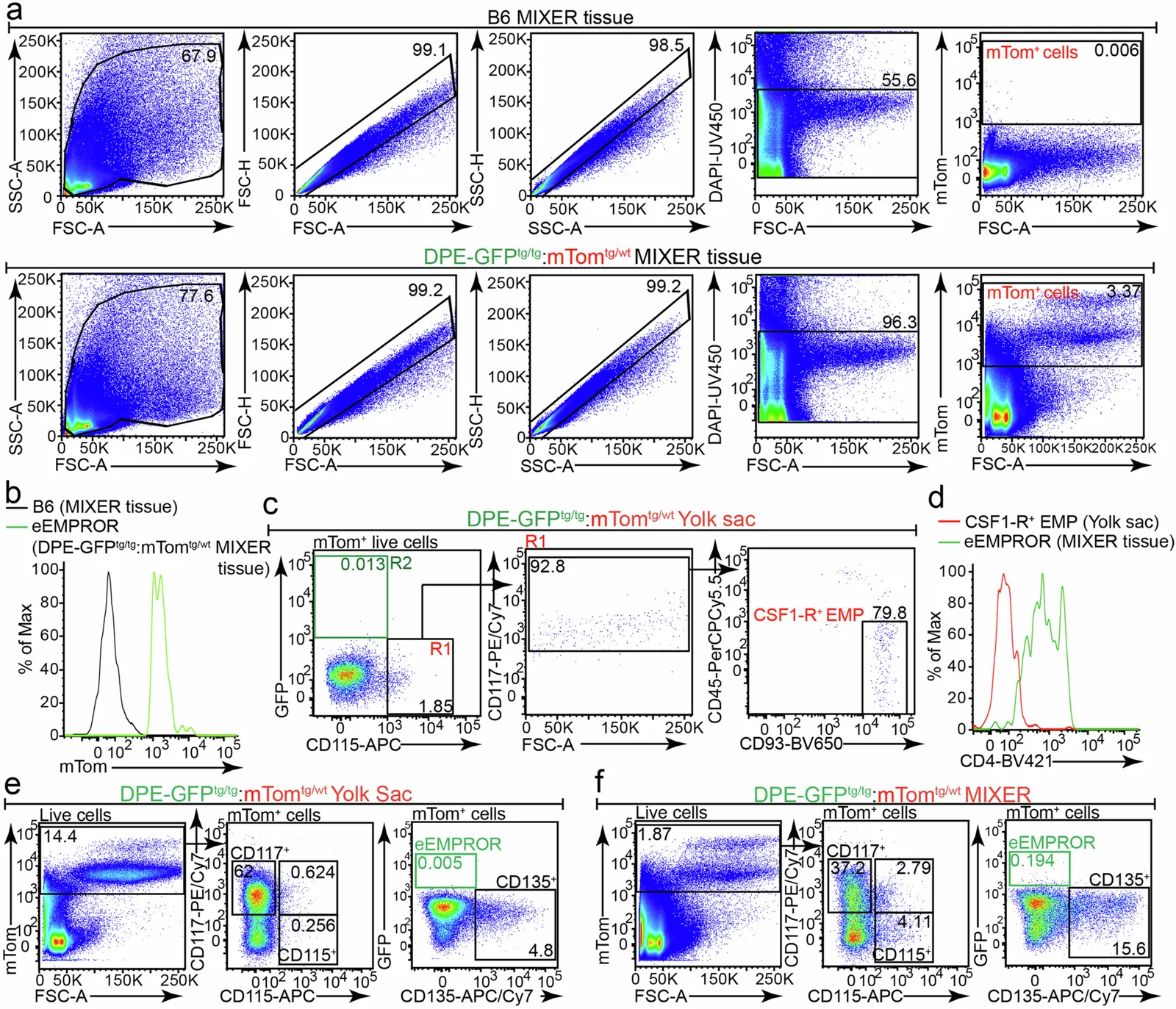
Identification of embryo-derived cells at the maternal-embryo interface. **a**, Flow-cytometric analysis of MIXER tissue isolated from either C57BL/6 or DPE-GFP^+/+^:mTom^+/−^ conceptuses depicting the gating strategy for mTom^+^ cells. Representative flow plots from n = 5 independent litters (6–11 embryos/litter) from five separate timed-mating pairs from three independent experiments are shown. The embryos were staged as E8.5 with 6–9 somite pairs. **b**, Histogram plots indicating the expression of mTom by eEMPROR cells identified in the MIXER tissue of DPE-GFP^+/+^:mTom^+/−^ conceptuses. Representative flow plots from n = 5 independent litters (6–11 embryos/litter) from five separate timed-mating pairs from three independent experiments are shown. The embryos were staged as E8.5 with 6–9 somite pairs; **c**, Flow cytometric analysis highlighting the presence of CSF1-R^+^ EMP but not eEMPROR cells within the yolk sac at E8.5; **d**, Histogram plots showing the lack of CD4 expression on CSF1-R^+^ EMP within the yolk sac as compared to MIXER tissue-derived eEMPROR cells. Panels **c** and **d**, data representative of n = 4 independent litters (6–11 embryos/litter) from four separate timed-mating pairs from two independent experiments. The embryos were staged as E8.5 with 6–8 somite pairs. Please note that the histogram plots depicting the expression of CD4 in eEMPROR cells in both Fig. 4e and Extended Data Fig. 3d are the same and the data is obtained from the MIXER tissue as the yolk sac is devoid of eEMPROR cells. **e** and **f**, Flow cytometric analysis of various lineage markers expressed by mTom^+^ cells present within the yolk sac and the MIXER tissue, respectively. Representative flow plots from n = 6 independent litters (6–11 embryos/litter) from six separate timed-mating pairs from three independent experiments are shown. The embryos were staged as E8.5 with 6–9 somite pairs.

Given our identification of mature blood cells of embryonic origin within MIXER before the establishment of a robust embryonic–extraembryonic circulation, we next sought to test its hematopoietic capacity directly. In parallel, we aimed to define the hematopoietic progenitor populations present in MIXER and the yolk sac, including EMPROR cells, in greater detail. To accomplish this, we generated conceptuses from female DPE-GFP mice crossed with male DPE-GFP:mT/mG mice (Fig. 3a), a strategy that allowed clear discrimination between maternal (mTom^−^), embryonic (mTom^+^) and eEMPROR cells (GFP^+^mTom^+^). Briefly, in the transgenic DPE-GFP mouse strain^41,47–49^, CD4 regulatory elements direct the expression of GFP in T cells^48^, perivascular macrophages (PVMs)^48^ and plasmacytoid dendritic cells^50^ (Methods). Furthermore, the expression of GFP in these mice allowed for the identification of EMPROR cells in adult bone marrow^41^. As observed in our β-actin-Cre × mT/mG analyses, this approach enabled unambiguous discrimination between the embryonic and maternal compartments (Extended Data Fig. 3a,b).

Initially, we sorted mTom^+^ embryonic cells from MIXER and yolk sac tissues and cocultured them with OP9 stromal cells in medium supplemented with stem cell factor (SCF), interleukin-3 (IL-3), IL-6, erythropoietin (EPO), thrombopoietin (TPO), granulocyte–macrophage colony-stimulating factor (GM-CSF) and macrophage colony-stimulating factor (M-CSF). Flow cytometric analysis and Giemsa staining demonstrated robust generation of erythroid, myeloid and megakaryocytic lineages from both tissues (Fig. 3b–g). Notably, most leukocytes derived from MIXER cultures expressed CD31 (Fig. 3d), similar to those produced from yolk sac progenitors (Fig. 3e), suggesting a potential hemangiogenic origin. These findings indicate that MIXER harbors functionally competent hematopoietic progenitors capable of multilineage differentiation, thereby establishing the maternal–fetal interface as an active site of embryonic hematopoiesis alongside the yolk sac.

### eEMPROR cells preferentially reside within the MIXER tissue

**Fig. 4:**
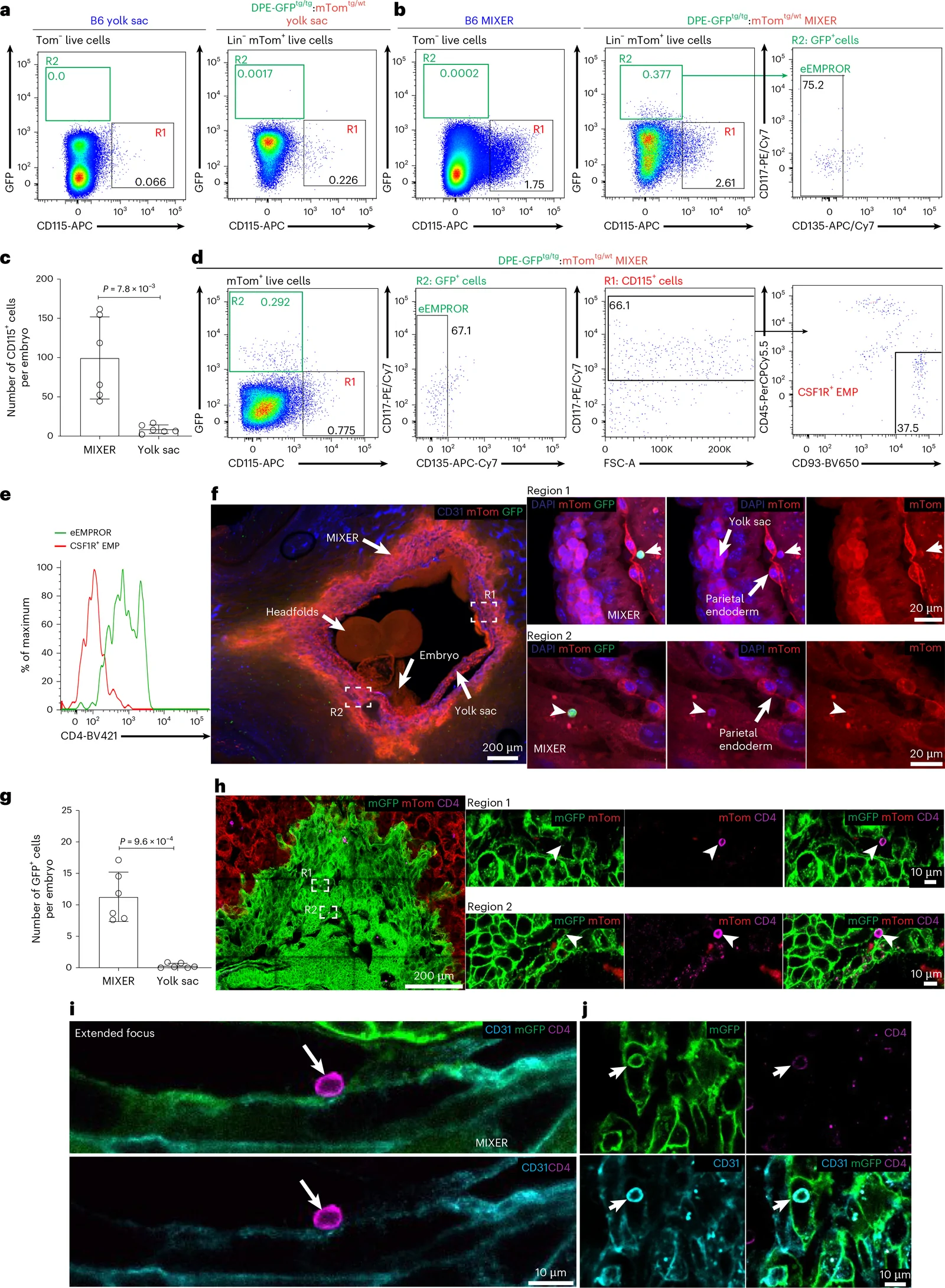
eEMPROR cells arise within the MIXER tissue. **a**,**b**, Flow cytometric analysis of the yolk sac (**a**) and MIXER tissue (**b**) from E8.5 embryos, identifying the presence of CD115^+^ cells in both tissues, along with distinct GFP^+^ cells within the MIXER tissue only. Lineage markers consist of CD3ε, Ly6G, NK1.1, CD19, Siglec-H and CD64. Representative flow plots from *n* = 5 independent litters (6–11 embryos per litter) from five separate timed mating pairs across three independent experiments are shown. The flow cytometry data presented are from eight embryos staged as E8.5 with six to nine somite pairs. **c**, Bar graph showing the number of CD115^+^ cells observed within the MIXER tissue and the yolk sac. Statistical significance was determined using a two-tailed unpaired *t-*test with Welch’s correction and a 95% confidence interval. Data are presented as mean ± s.d., with symbols representing individual data points from each litter. **d**, Flow cytometric analysis of the presence of CSF1R^+^ EMPs and eEMPROR cells within the E8.5 MIXER tissue. **e**, Histogram plots depicting CD4 expression on CSF1R^+^ EMPs and eEMPROR cells present within the E8.5 MIXER tissue. Data in **d** and **e** are representative of *n* = 4 independent litters (6–11 embryos per litter) from four separate timed mating pairs across two independent experiments. The embryos were staged as E8.5 with six to eight somite pairs. **f**, Representative confocal images of a section of the E8.5 conceptus, revealing the extent of extraembryonic tissue (MIXER) within the gravid uterus. Higher-magnification images highlight the presence of GFP^+^ cells within the MIXER tissue. The images are representative of *n* = 4 embryos from four independent timed mating pairs across three independent experiments. **g**, Bar graph showing the number of GFP^+^ cells observed within MIXER and the yolk sac, as determined by flow cytometry in **a** and **b**. Statistical significance was determined using a two-tailed unpaired *t*-test with Welch’s correction and a 95% confidence interval. Data are presented as mean ± s.d., with symbols representing individual data points from each litter. **h**, Confocal images highlighting the presence and localization of CD4^+^ cells within the MIXER tissue. **i**, Confocal images of the MIXER tissue highlighting the localization of CD4^+^ cells in the vicinity of endothelial cells. **j**, Confocal images of the MIXER tissue identifying the presence of mGFP^+^CD4^+^CD31^+^ cells close to endothelial cells. Panels **c** and **g** show cumulative data from three independent experiments, with *n* = 6 independent litters (46 embryos in total), normalized to the number of E8.5 embryos analyzed per experiment. All embryos were staged as E8.5 with six to nine somite pairs, except for one litter staged as E8.25–E8.5 with two to six somite pairs. Images in **h**, **i** and **j** are representative of four different embryos from two independent timed matings. Source data

Given the robust multilineage potential observed in MIXER cultures, we next sought to identify the specific progenitor populations responsible for this activity and to compare them directly with those present in the yolk sac. To this end, we performed flow cytometric analysis of microdissected MIXER, yolk sac and embryo proper tissues from E8.5 DPE-GFP × DPE-GFP:mT/mG conceptuses. Similar to the yolk sac (Fig. 4a), MIXER contained CD115^+^ hematopoietic precursor cells (Fig. 4b,c), including classical CSF1R^+^CD117^+^CD45ˡᵒCD93^+^ EMPs^14,51^ (termed here as CSF1R^+^ EMPs) (Fig. 4d). These canonical EMPs did not express CD4 in either MIXER (Fig. 4e) or the yolk sac (Extended Data Fig. 3c,d). Quantitatively, CSF1R^+^ cells were markedly more abundant in MIXER than in the yolk sac at this stage (Fig. 4c).

We also identified GFP^+^mTom^+^ cells within the MIXER tissue, but not in the yolk sac (Fig. 4a,b). This finding was further corroborated by laser scanning confocal microscopy, where we detected the presence of GFP^+^ cells within MIXER (Fig. 4f). These GFP^+^mTom^+^ cells also expressed CD4 (Fig. 4e), were robustly observed in MIXER—albeit at a lower frequency than CD115^+^ cells (Fig. 4g)—and shared phenotypic characteristics with adult EMPROR cells^41^. Therefore, we designated them eEMPROR cells (Fig. 4b). In addition to CSF1R^+^ EMPs and eEMPROR cells, we detected embryonically derived CD135^+^ and CD117^+^ hematopoietic progenitor cells within the MIXER tissue, similar to those found in the yolk sac (Extended Data Fig. 3e,f), further indicating an active hematopoietic environment.

**Extended Data Fig. 4:**
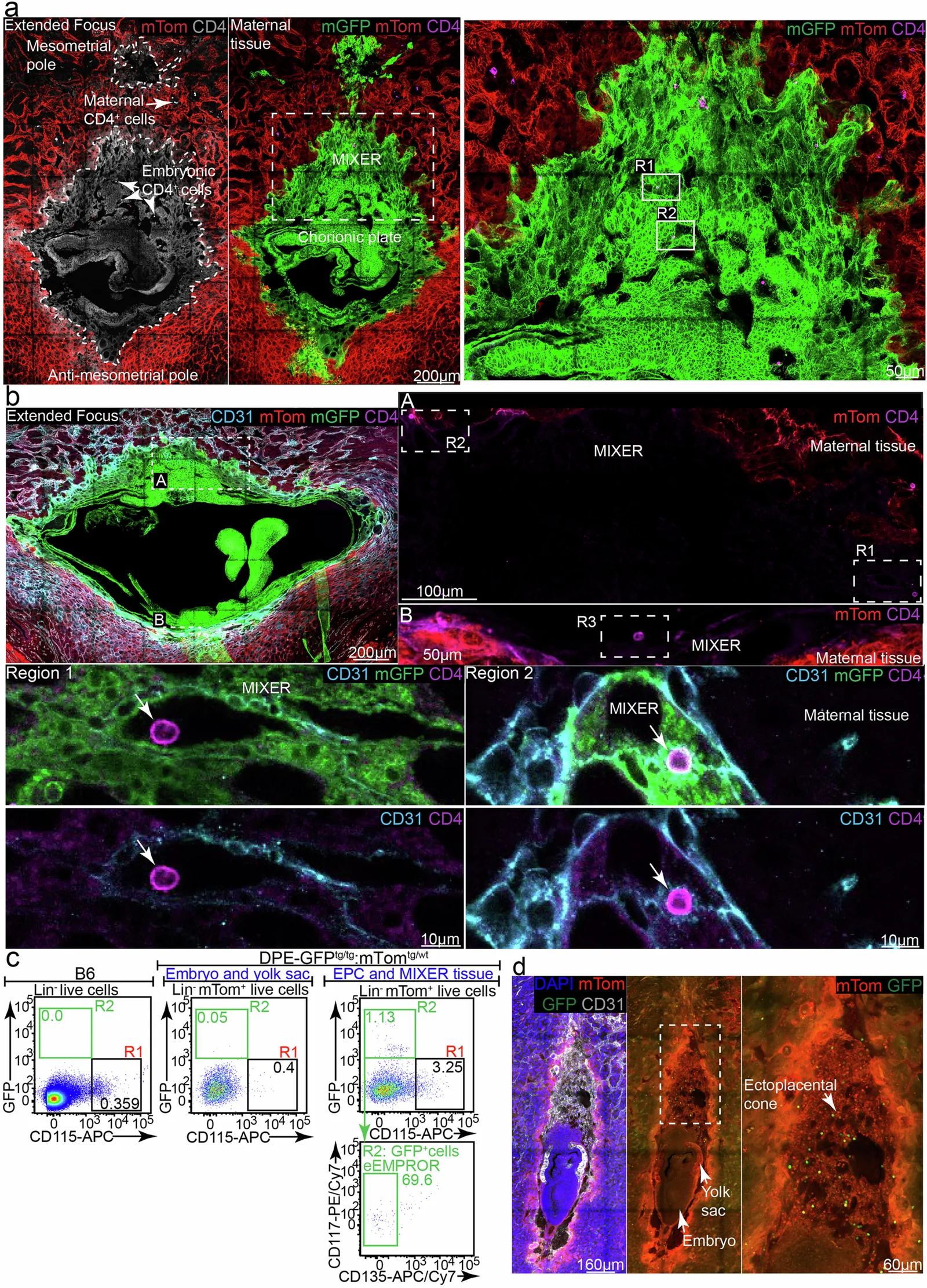
Characterization of CD4^+^ cells within the MIXER tissue. Confocal image of E8.5 conceptus from β-actin-Cre:mT/mG embryos highlighting the localization of: **a**, CD4^+^ cells within the MIXER (region demarcated) and maternal tissue (mTom^+^). The left panel image does not show mGFP to highlight the cells present within the MIXER tissue. The embryonic and extra-embryonic regions expressing mGFP can be observed within the corresponding middle panel, while the right panel shows a magnified image of the tissue with regions 1 and 2. The magnified images for regions 1 and 2 is presented in Fig. 4h; **b**, Confocal imaging of immunostained sections of the conceptus, highlighting the presence of a few embryonic (mGFP^+^mTom^−^) CD4^+^ cells in close proximity to CD31^+^ cells within the MIXER tissue. Subpanel **a** and **b** images on the right show the CD4 signal in the absence of mGFP overlay. The magnified images for regions 1 and 2 are presented below, while the data for region 3 are presented in Fig. 4i. Panels **a** and **b**, data representative of 4 different embryos from 2 independent timed matings. **c**, Flow cytometric analysis at E7.5 confirms the presence of mTom^+^GFP^+^ cells within the MIXER tissue of DPE-GFP:mT/mG embryos. **d**, Confocal imaging of gravid uterus at E7.5, highlighting the different anatomical compartments of the developing conceptus, including MIXER tissue. The images reveal the presence of mTom^+^CD31^+^ and mTom^+^GFP^+^ cells in the ectoplacental region of the MIXER tissue and mTom^+^CD31^+^ cells in the developing yolk-sac.

To corroborate the presence of CD4^+^ progenitor cells in addition to the DPE-GFP transgene and to examine their precise localization, tissue sections collected at E8.5 from β-actin-Cre × mT/mG timed matings were immunostained for CD4 and subsequently analyzed by confocal imaging. CD4^+^ cells were present within various regions of MIXER (Fig. 4h and Extended Data Fig. 4a,b) and were consistently found in close proximity to CD31^+^ endothelial cells (Fig. 4i and Extended Data Fig. 4b). On rare occasions, we observed CD4^+^CD31^+^ double-positive cells within the MIXER tissue, highlighting the possibility of their recent emergence from hemogenic endothelial cells (Fig. 4j).

Together, these findings identify MIXER as a new site containing both classical CSF1R^+^ EMPs and a distinct eEMPROR cell population, revealing the coexistence of two EMP lineages at the maternal–fetal interface during early hematopoiesis.

### MIXER tissue displays hematopoietic features as early as E7.5

**Fig. 5:**
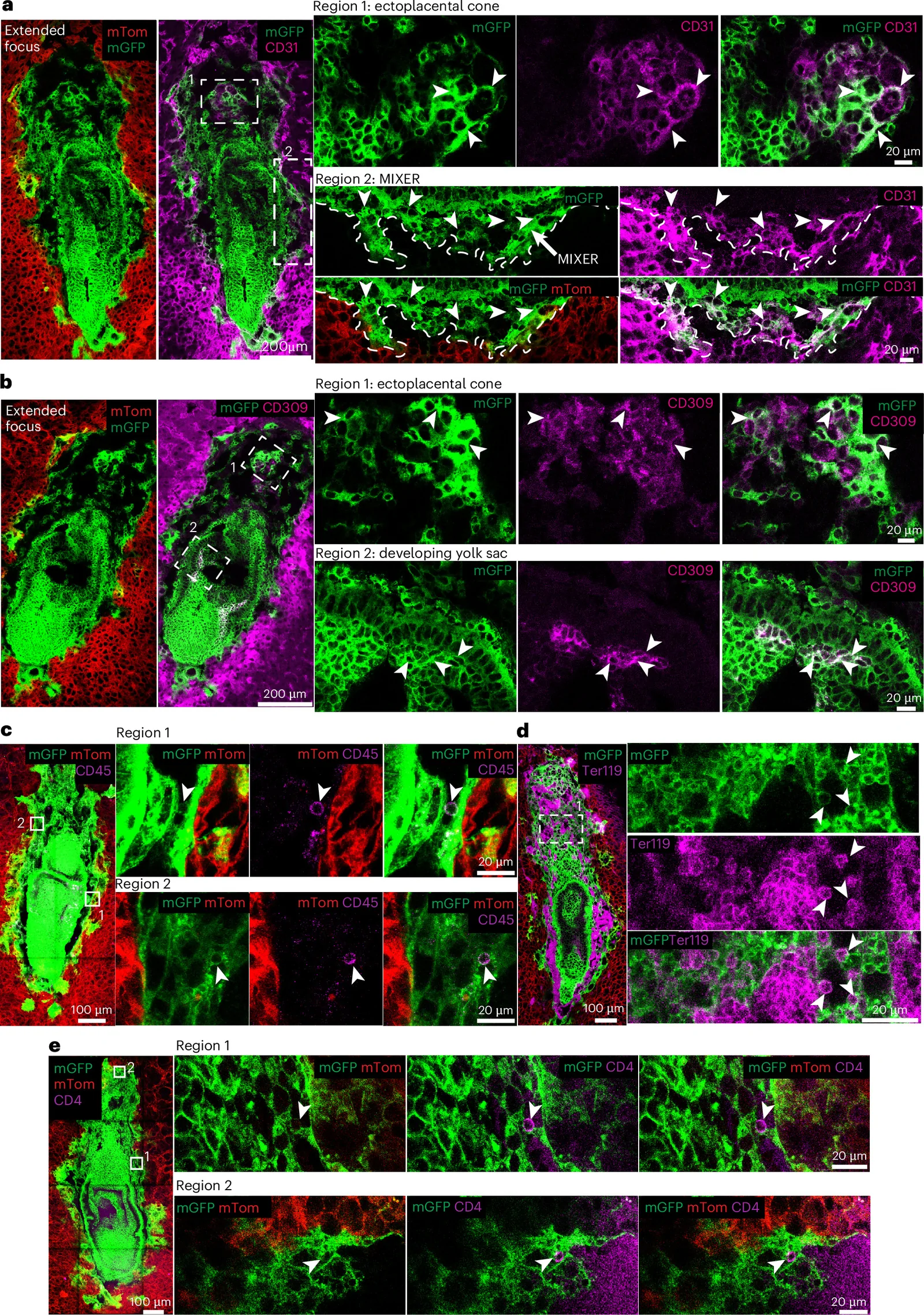
Early hematopoiesis within the MIXER tissue. **a**, Confocal imaging of an E7.5 conceptus highlighting the expression of CD31 within mGFP^+^ cells in the ectoplacental cone (region 1, arrowheads) and the MIXER tissue at the periphery (region 2, arrowheads). Images are representative of nine embryos from six timed mating experiments. **b**, Serial sections of the above embryo demonstrating the expression of CD309 (FLK1) within the ectoplacental cone (region 1, arrowheads) and the developing yolk sac (region 2, arrowheads). Images are representative of seven embryos from five timed mating experiments. **c**–**e**, Confocal images highlighting the expression of hematopoietic markers by mGFP^+^ cells within MIXER tissue: CD45 (**c**), Ter119 (**d**) and CD4 (**e**). Images in **c** and **d** are representative of four embryos from four timed mating experiments. In **e**, the images are representative of ten embryos from five timed mating experiments.

Because yolk sac hematopoiesis can be observed as early as E7.5, we next asked whether MIXER also exhibits hematopoietic features at this earlier developmental stage. To address this question, β-actin-Cre male mice were paired with mT/mG female reporter mice for timed mating (Extended Data Fig. 1), enabling unambiguous discrimination between maternally derived mTom^+^ cells and embryonically derived mGFP^+^ cells. Whole gravid uteri were sectioned and analyzed by confocal laser scanning microscopy. Similar to the findings at E8.5, double-transgenic conceptuses at E7.5 displayed a clear separation of maternal and embryonic compartments, with embryonic mGFP^+^ cells readily identifiable within the MIXER region (Fig. 5a, left).

To determine whether the MIXER tissue already contains endothelial elements compatible with early hematopoietic activity, cryosections were costained for CD31. Confocal imaging of these sections revealed the presence of CD31^+^ mGFP^+^ embryonic endothelial cells distributed within the MIXER tissue (Fig. 5a, right). We next examined the expression of CD309 (VEGFR2/KDR/FLK1), a marker associated with early hemogenic endothelium and hemangioblastic populations that participate in the first waves of embryonic blood formation^52–54^. CD309^+^ cells were readily detected within the MIXER region (Fig. 5b), supporting the presence of a local endothelial microenvironment permissive for early hematopoietic development.

We then asked whether differentiated embryonic hematopoietic cells were already present within this compartment. Costaining with CD45 identified embryonic leukocytes within MIXER (Fig. 5c), whereas Ter119 staining demonstrated abundant embryonic erythroid cells throughout the tissue (Fig. 5d). Together, these findings indicate that MIXER harbors both endothelial and hematopoietic components at E7.5, consistent with hematopoietic activity occurring at the maternal–fetal interface during the earliest stages of development.

In addition, CD4^+^ mGFP^+^ cells were detectable within the MIXER tissue at E7.5, suggesting that eEMPROR cells may emerge during this early developmental window (Fig. 5e). To validate this observation quantitatively and in a different model system, we performed flow cytometric analyses of microdissected MIXER, yolk sac and embryo tissues from E7.5 DPE-GFP:mT/mG conceptuses. These analyses identified GFP^+^ eEMPROR cells selectively within MIXER, whereas corresponding yolk sac preparations lacked this population (Extended Data Fig. 4c). In parallel, MIXER also contained abundant CD115^+^ hematopoietic progenitor cells (Extended Data Fig. 4c). Confocal imaging independently confirmed the presence of GFP^+^ cells throughout the MIXER region (Extended Data Fig. 4d). Together, these findings indicate that both canonical CD115^+^ progenitors and eEMPROR cells are already established within the maternal–fetal interface by E7.5, placing MIXER among the earliest embryonic sites associated with erythro-myeloid development.

### eEMPROR cells in later embryonic development

**Fig. 6:**
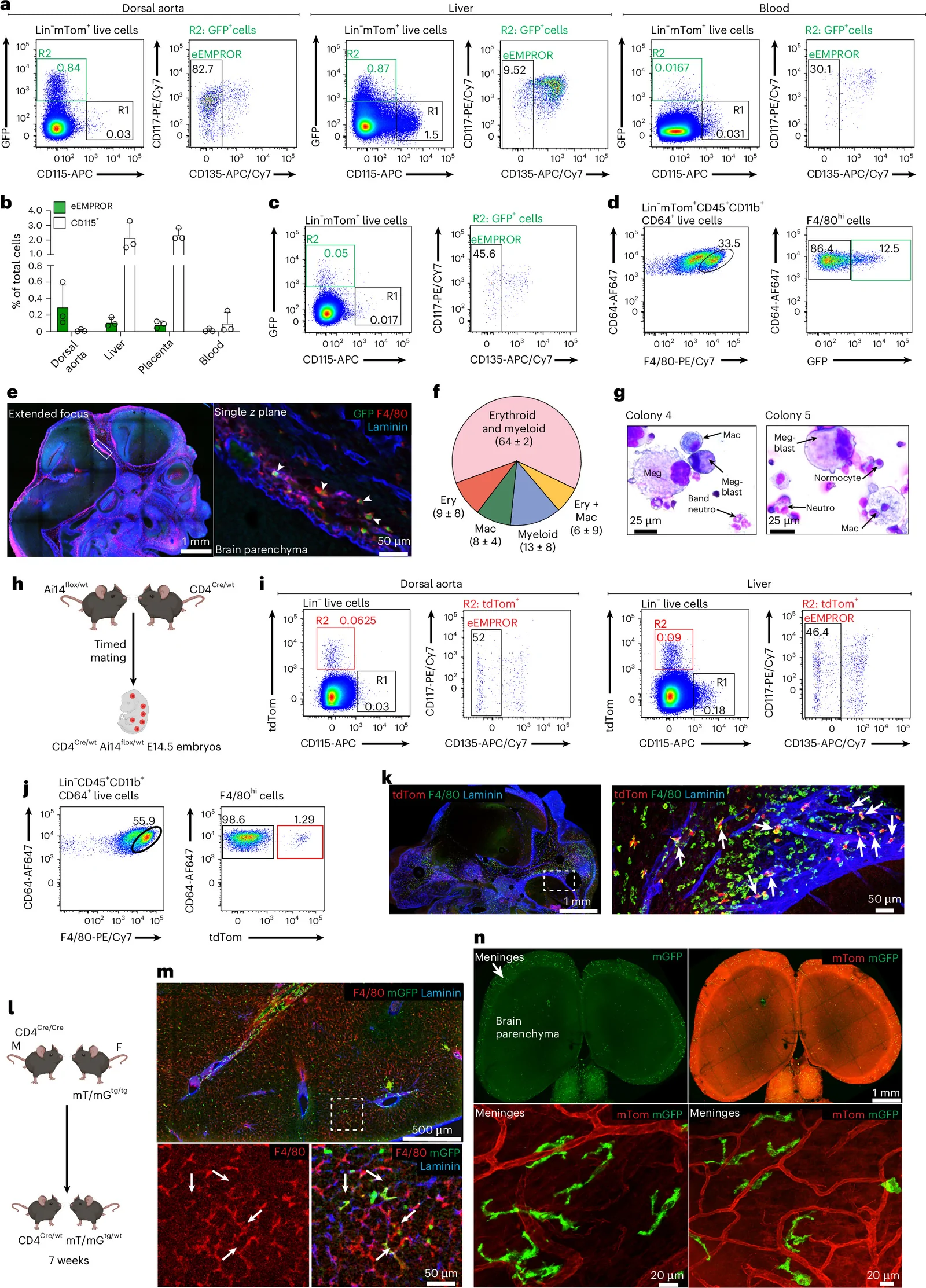
Embryonic and postnatal development of the CD4 lineage. **a**, Flow cytometric analysis of the dorsal aorta, liver and blood of DPE-GFP^tg^^/^^tg^:mT/mG^tg^^/wt^ E14.5 embryos. The data reveal the presence of eEMPROR cells and lin^−^GFP^−^CD115^+^ precursors within these tissues. Representative flow plots from one of four independent experiments are shown. **b**, Quantification of eEMPROR cells and CD115^+^ precursors within various hematopoietic organs of DPE-GFP^tg^^/^^tg^:mT/mG^tg^^/wt^ E14.5 embryos. Data shown are the cumulative results from three independent experiments. Bars with error bars represent mean ± s.d. **c**, Flow cytometric analysis of E14.5 embryo heads revealing the presence of eEMPROR cells. Representative flow plots from one of three independent experiments are shown. **d**, Flow cytometric analysis of fetal heads identifying the presence of GFP^+^ macrophages. Representative flow plots from one of four independent timed mating experiments are shown. **e**, Confocal images of immunostained E14.5 embryo head tissue showing GFP^+^F4/80^+^ macrophages (white arrowheads) around the brain parenchyma. Representative images of *n* = 3 E14.5 embryos collected from three independent timed mating pairs across three independent experiments are shown. **f**, Pie chart depicting the proportion of macrophage (Mac), myeloid (macrophage and neutrophil), erythroid and macrophage (Ery + Mac), erythroid (Ery) and erythro-myeloid (erythroid + myeloid; erythroid with macrophage and neutrophil lineages) colonies generated following the culture of sorted eEMPROR cells from the E14.5 fetal liver in MethoCult GF M3434 containing transferrin, SCF, IL-3, IL-6 and EPO, further supplemented with TPO, GM-CSF and M-CSF. Data are the cumulative results of 62 colonies from three independent sorting and culture experiments. The values represent the mean percentage of colonies ± s.d. for each population. **g**, Giemsa staining of colonies depicting erythroid as well as myeloid lineage potential generated following the culture of sorted eEMPROR cells in MethoCult GF M3434 containing transferrin, SCF, IL-3, IL-6 and EPO, further supplemented with TPO, GM-CSF and M-CSF. Data represent 2 of 21 colonies across three independent sorting and culture experiments. Mac, macrophage; Meg-blast, megakaryoblast; Meg, megakaryocyte; Neutro, neutrophil; Band neutro, band neutrophil. **h**, Schematic representation of the timed mating strategy for CD4-Cre:Ai14 lineage-tracing experiments. **i**, Flow cytometric analysis of the dorsal aorta and liver of tdTom^+^ E14.5 embryos from the CD4-Cre:Ai14 timed mating studies. The data reveal the presence of lin^−^tdTom^+^CD115^−^CD117^+^ eEMPROR cells and lin^−^ tdTom^−^CD115^+^ precursors within these tissues. Representative flow plots from one of four independent experiments are shown. **j**, Flow cytometric analysis of the fetal head identifying the presence of tdTom^+^ macrophages. Representative flow plots from one of four independent experiments are shown. Lineage markers in **a**, **c** and **i** include CD3ε, Ly6G, NK1.1, CD19, Siglec-H and CD64. **k**, Confocal images of immunostained E14.5 embryo heads showing tdTom^+^F4/80^+^ macrophages (white arrows) around the vasculature. Representative images were from *n* = 3 E14.5 embryos collected across three independent experiments. **l**, Schematic detailing the timed mating setup. **m**, Confocal images of liver tissue from 7-week-old CD4^Cre/wt^ mT/mG^tg/wt^ mice highlighting the presence of numerous mGFP^+^ cells along the blood vessels and a few mGFP^+^ macrophages (white arrows) within the interstitium (magnified images). Most Kupffer cells were observed to be mGFP^−^. Images are representative of four mice from three independent experiments. **n**, Images highlighting the presence of mGFP^+^ cells within the meninges of 7-week-old CD4^Cre/wt^ mT/mG^tg^^/wt^ mice. The bottom images highlight the localization of mGFP^+^ cells along the meningeal vasculature. Images are representative of five mice from four independent experiments. Icons in **h** and **l** created in BioRender; Jain, R. https://biorender.com/f6aluj6 (2026). Source data

**Extended Data Fig. 5:**
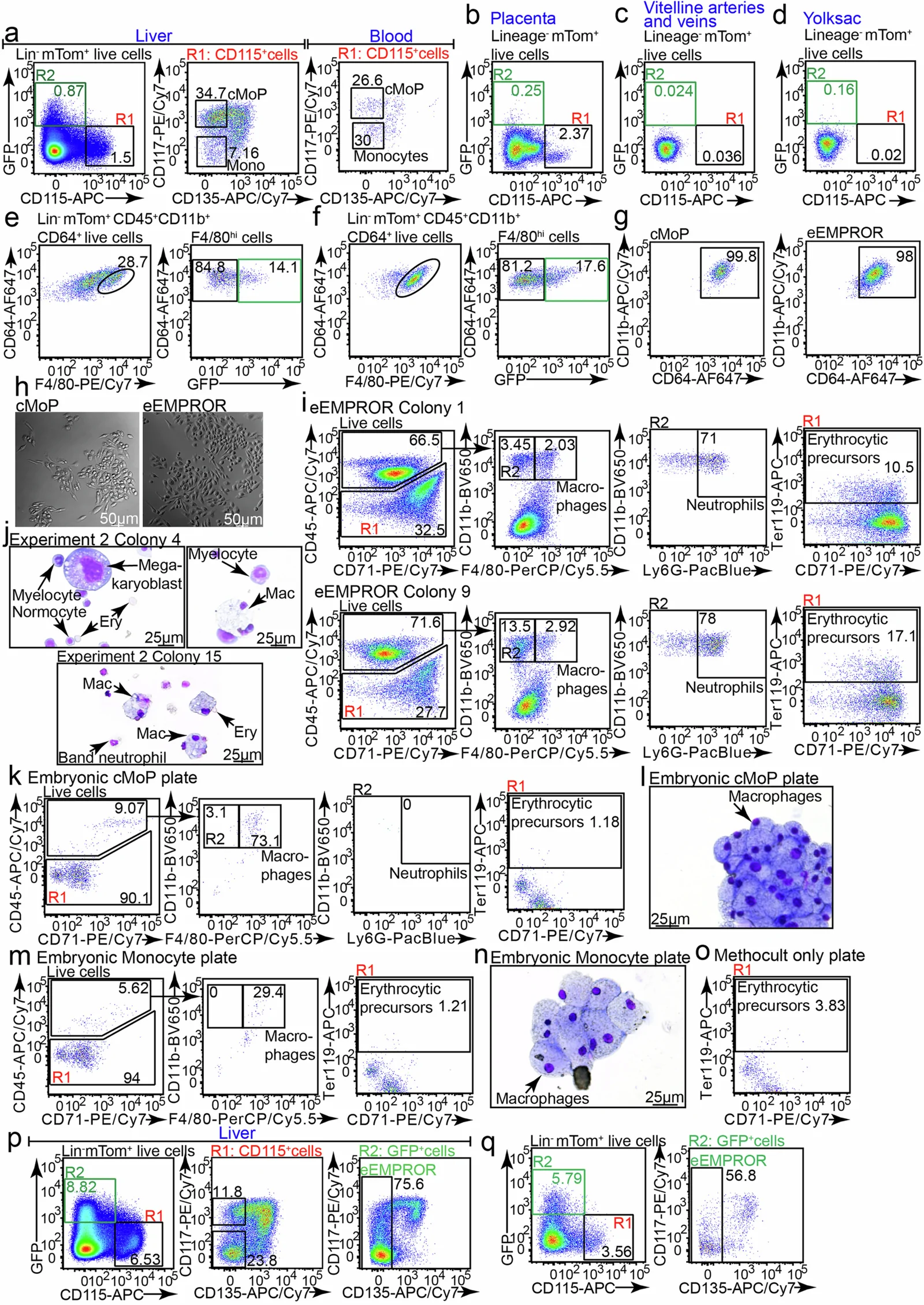
Development of eEMPROR cells and macrophage precursors. **a**, Flow cytometric analysis of E14.5 fetal liver and blood showing the presence of CD115^+^ cMoP and monocytes. Representative flow plots from one of four independent experiments are shown; **b**, Flow cytometric analysis of E14.5 fetal placenta depicting the presence of a few GFP^+^ precursors. Representative flow plots from one of four independent experiments are shown; **c**, Flow cytometric analysis of E14.5 vitelline arteries and veins displaying the presence of a few GFP^+^ precursors. Representative flow plots from one of four independent experiments are shown; **d**, Flow cytometric analysis of E14.5 yolk sac indicating the absence of GFP^+^ precursors. Representative flow plots from one of four independent experiments are shown. Lineage markers for panel **a**-**d** consist of CD3e, Ly6G, NK1.1, CD19, Siglec-H, and CD64. **e**, Flow cytometric analysis of fetal skin demonstrating the presence of GFP^+^ macrophages. Representative flow plots from one of two independent experiments are shown; **f**, Flow cytometric analysis of E14.5 yolk sac detailing the presence of both GFP^−^ and GFP^+^ macrophages. Representative flow plots from one of three independent experiments are shown; **g**, Flow cytometric analysis illustrating the development of CD11b^+^CD64^+^ macrophages from sorted cMoP and eEMPROR cells when cultured with 50 ng/ml M-CSF. Representative flow plots from one of three independent sort and culture experiments are shown; **h**, Confocal imaging of macrophages obtained from *in vitro* culture of sorted embryonic cMoP and eEMPROR cells in RPMI medium containing M-CSF. Representative images from one of two independent sort and culture experiments are shown; **i**, Flow cytometric analysis of two representative colonies depicting erythroid and myeloid (macrophage and neutrophil) lineage potential of sorted eEMPROR cells in MethoCult™ GF M3434 containing transferrin, SCF, IL-3, IL-6, EPO, TPO, GM-CSF, and M-CSF (see Methods for details). Data representative of 62 colonies from three independent sort and culture experiments; **j**, Giemsa staining of two independent colonies highlighting erythroid and myeloid lineage potential of sorted eEMPROR cells in MethoCult™ GF M3434 containing transferrin, SCF, IL-3, IL-6, EPO, and supplemented with TPO, GM-CSF, and M-CSF. Data represent images from two of 21 independent colonies from one of the three independent sort and culture experiments. Mac-Macrophages, Ery-Erythrocytes; **k**, Flow cytometric analysis of cells demonstrating the macrophage differentiation potential of sorted embryonic cMoP in MethoCult™ GF M3434 containing SCF, IL-3, IL-6, EPO, and supplemented with TPO, GM-CSF, and M-CSF. Data representative of colonies from three independent sort and culture experiments; **l**, Giemsa staining of cells obtained from cMoP culture displaying macrophage lineage potential. Data representative of one of three independent sort and culture experiments; **m**, Flow cytometric analysis of cells recovered from monocyte cultures revealing the restricted macrophage potential of embryonic monocytes post-culturing in MethoCult™ GF M3434 containing transferrin, SCF, IL-3, IL-6, EPO, and supplemented with TPO, GM-CSF, and M-CSF. Data representative of one of three independent sort and culture experiments; **n**, Giemsa staining of cells delineates the macrophage lineage potential generated post-culture of sorted embryonic monocytes in MethoCult™ GF M3434 containing transferrin, SCF, IL-3, IL-6, EPO, and supplemented with TPO, GM-CSF, and M-CSF. Data representative of one of three independent sort and culture experiments. Both embryonic cMoP and monocytes lack erythroid and neutrophil lineage potential under these conditions; **o**, Flow cytometric analysis of MethoCult™ GF M3434 without any cells, highlighting the background signal present in the medium. Data is from a single experiment. **p**, Flow cytometric analysis of fetal livers from E19.5 embryos exhibiting the presence of eEMPROR cells. The presence of lin^−^GFP^+^CD115^−^CD117^+^CD135^+^ and lin^−^GFP^−^CD115^+^ precursors can also be detected within the tissue. Lineage markers consist of CD3e, Ly6G, NK1.1, CD19, Siglec-H, and CD64. Representative flow plots from one of three independent experiments are shown. **q**, Flow cytometric analysis of developing bone marrow from E19.5 embryos. Lineage markers consist of CD3e, Ly6G, NK1.1, CD19, Siglec-H, and CD64. Representative flow plots from one of three independent experiments are shown.

Having identified both CSF1R^+^ EMPs and a distinct eEMPROR cell population within MIXER at E8.5, as well as CSF1R^+^ cells and eEMPROR cells at E7.5, we next asked whether these cells contribute to hematopoiesis at later developmental stages akin to classical EMP cells. Because hematopoietic activity transitions through multiple tissues during embryogenesis, we examined several organ sites at E14.5. In line with the literature^55^, we detected a robust population of lin^−^GFP^−^CD115^+^ cells within the fetal liver and placenta (Fig. 6a,b and Extended Data Fig. 5a,b), which included the embryonic common monocyte progenitors (cMoPs) and monocytes^55^ (Extended Data Fig. 5a). Strikingly, the dorsal aorta showed the highest frequency of eEMPROR cells (Fig. 6a,b). Notably, however, the placental labyrinth—one of the major sites of hematopoiesis at this stage^56^—harbored very few GFP^+^ cells (Extended Data Fig. 5b). Similarly, the vitelline arteries and veins were devoid of eEMPROR cells (Extended Data Fig. 5c), as were the E14.5 yolk sacs (Extended Data Fig. 5d).

To assess whether eEMPROR cells may also give rise to GFP^+^ macrophages, flow cytometry and confocal imaging were performed on E14.5 embryos. eEMPROR cells, as well as GFP^+^CD64^+^F4/80^+^ macrophages, were found within the embryo heads (Fig. 6c,d). Many of these macrophages localized to the perivascular space (Fig. 6e). Similarly, GFP^+^ macrophages were identified within the skin (Extended Data Fig. 5e). Collectively, these findings suggest that eEMPROR cells migrate to peripheral tissues, where they may differentiate into GFP^+^ PVMs. Interestingly, yolk sacs harbored GFP^+^ macrophages (Extended Data Fig. 5f).

To formally test their ability to generate macrophages in vitro, we sorted cMoPs and eEMPROR cells from E14.5 embryonic livers and cultured them in the presence of M-CSF. Both cell populations produced macrophages (Extended Data Fig. 5g,h). In single-cell-derived colony-forming assays^41^, eEMPROR cells gave rise to colonies consisting of macrophage, neutrophil, megakaryocytic and erythroid lineages (Fig. 6f,g and Extended Data Fig. 5i,j), highlighting their erythro-myeloid potential in vitro. As expected, monocytes did not generate colonies, and cMoP colonies were restricted to the monocyte–macrophage lineage (Extended Data Fig. 5k–o).

Finally, we assessed whether eEMPROR cells persist into late gestation. Flow cytometry at E19.5 revealed a robust population of eEMPROR cells in both the fetal liver and developing bone marrow (Extended Data Fig. 5p,q), indicating continued migration and residence within prenatal hematopoietic tissues.

### CD4-lineage tracing identifies embryonic macrophages

Having identified the eEMPROR cell population, which is phenotypically distinct from classical CSF1R^+^ EMPs and enriched within the MIXER tissue, we next asked whether CD4 expression marks a bona fide embryonic progenitor lineage that contributes to macrophage development in vivo. To address this question, we used both constitutive and inducible CD4-Cre lineage-tracing systems.

**Extended Data Fig. 6:**
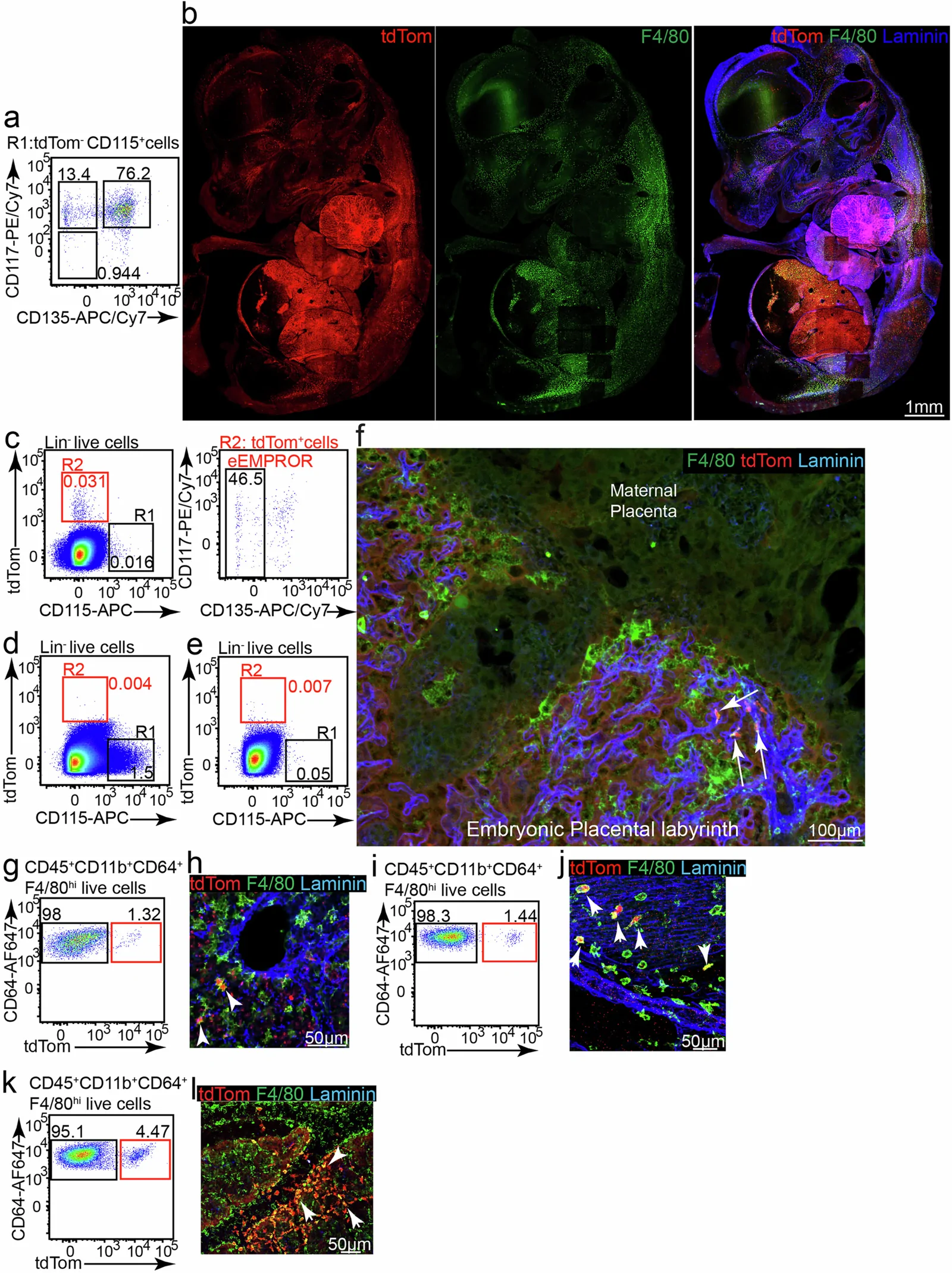
Development of eEMPROR cells and macrophage precursors in CD4Cre x Ai14 mice. **a**, Flow cytometric analysis of the liver of tdTom^+^ E14.5 embryos from CD4Cre:Ai14 timed mating studies presented in Fig. 6i**, gate R1**, the data highlight the presence of cMoP and monocytes within the tdTom^−^CD115^+^ gate. Representative flow plots from one of four independent experiments are shown; **b**, Confocal imaging of tdTom^+^ E14.5 embryo obtained from timed mating of CD4Cre and Ai14 mice. The tiled images show the distribution of tdTom^+^ and F4/80^+^ cells within various tissues, highlighted by laminin staining. Data is representative of three independent timed matings; **c**, Flow cytometric analysis of E14.5 embryo head revealing the presence of tdTom^+^CD115^−^CD117^+^ eEMPROR cells. Representative flow plots from one of four independent experiments are shown; **d**, Flow cytometric analysis of E14.5 fetal placenta depicting the absence of tdTom^+^ precursors. A representative flow plot from one of four independent experiments is shown; **e**, Flow cytometric analysis of E14.5 yolk sac indicating the absence of tdTom^+^ precursors. A representative flow plot from one of four independent experiments is shown. Lineage markers for panel **c**, **d** and **e** consist of CD3e, Ly6G, NK1.1, CD19, Siglec-H, and CD64; **f**, Confocal images highlight the presence of rare tdTom^+^ macrophages (only section with a few labelled cells) from the CD4 lineage within the placenta of E14.5 embryos. Data representative of two independent timed mating experiments. **g**, Flow cytometric analysis of E14.5 liver detailing the presence of both tdTom^−^ and tdTom^+^ macrophages. A representative flow plot from one of four independent experiments is shown; **h**, Confocal image marking the presence of tdTom^+^F4/80^+^ macrophages (white arrowheads) within fetal liver; **i**, Flow cytometric analysis of E14.5 skin detailing the presence of both tdTom^−^ and tdTom^+^ macrophages. A representative flow plot from one of four independent experiments is shown; **j**, Confocal microscopic image highlighting the presence of tdTom^+^F4/80^+^ macrophages (white arrowheads) within E14.5 fetal skin; **k**, Flow cytometric analysis of E14.5 yolk sac marking the presence of both tdTom^−^ and tdTom^+^ macrophages. A representative flow plot from one of four independent experiments is shown; **l**, Confocal microscopic image highlighting the presence of tdTom^+^F4/80^+^ macrophages (white arrowheads) within E14.5 yolk sac. Panels **h**, **j**, and **l**, images representative of three independent experiments.

We first performed timed matings by pairing CD4-Cre transgenic mice with Ai14 mice^57,58^ (Fig. 6h), in which tdTomato (tdTom^+^) irreversibly labels cells with a history of CD4 promoter activity. If eEMPROR cells indeed represent a CD4-marked EMP population, we reasoned that tdTom^+^ progenitors and tdTom^+^ macrophage descendants should remain detectable at later gestational stages. Consistent with this prediction, E14.5 embryos contained a distinct population of lin^−^CD115^−^CD117^+^tdTom^+^ progenitor cells within the dorsal aorta and fetal liver (Fig. 6i), closely paralleling the anatomical distribution previously observed for eEMPROR cells in DPE-GFP embryos. By contrast, CD115^+^ cMoP and monocyte populations were uniformly tdTom^−^ (Fig. 6i and Extended Data Fig. 6a), indicating that conventional monocyte-derived macrophage precursors do not share a history of CD4 promoter activity.

Confocal imaging further revealed that tdTom^+^ macrophages constitute a discrete subset of F4/80^+^ cells throughout the embryo (Extended Data Fig. 6b), consistent with the existence of a macrophage population derived from transient CD4-expressing embryonic progenitors. Spatial analysis additionally identified tdTom^+^ eEMPROR cells within the embryo head (Extended Data Fig. 6c), whereas comparable tdTom^+^ progenitors were not detected in either the placenta or yolk sac (Extended Data Fig. 6d,e). This distribution aligns with our earlier observations that CD4^+^ eEMPROR cells are selectively associated with MIXER and are not a prominent component of conventional extraembryonic hematopoietic tissues. Consistent with this, only rare tdTom^+^F4/80^+^ macrophages were observed within the placenta (Extended Data Fig. 6f), suggesting that CD4-marked progenitors make only a minimal contribution to Hofbauer-like macrophage populations.

Across tissues, tdTom^+^F4/80^+^ macrophages were reproducibly detected in the head and meninges (Fig. 6j,k), fetal liver (Extended Data Fig. 6g,h), skin (Extended Data Fig. 6i,j) and yolk sac (Extended Data Fig. 6k,l). Taken together, these findings demonstrate that CD4 marks eEMPROR cells and their macrophage progeny, in contrast to CD115, which selectively labels classical monocyte-derived lineages.

To determine whether the CD4-marked embryonic lineage contributes to tissue-resident macrophage populations beyond gestation, we next analyzed liver and brain tissues from 7-week-old CD4^Cre/wt^mT/mG^tg/wt^ mice, in which cells with a history of CD4 promoter activity are constitutively labeled by mGFP expression (Fig. 6l). In the young adult liver, mGFP^+^ macrophages constituted a small but readily identifiable subset of the total macrophage compartment (Fig. 6m), indicating the persistence of CD4-lineage descendants after birth. Analysis of the young adult brain revealed a markedly restricted pattern of contribution. Here, mGFP^+^ CD4-lineage macrophages were detected almost exclusively within the meningeal PVM compartment, whereas only minimal labeling was observed among parenchymal macrophages (Fig. 6n). Notably, the microglial population was entirely devoid of mGFP labeling (Fig. 6n). These findings indicate that CD4-marked embryonic progenitors give rise to a spatially selective subset of tissue macrophages, with a particularly prominent contribution to the meningeal perivascular niche.

### CD4^+^ progenitors contribute to fetal and postnatal macrophages

**Fig. 7:**
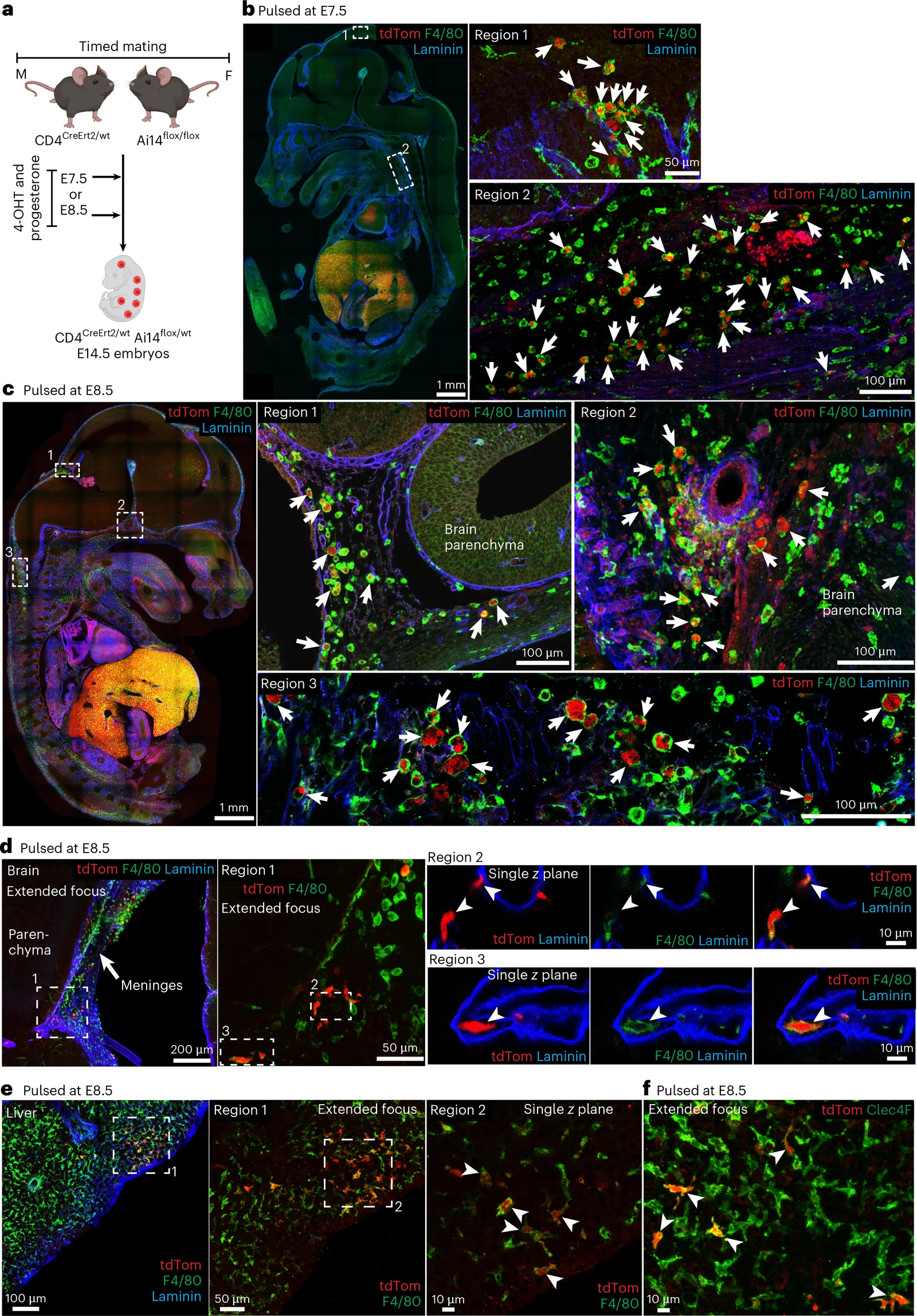
CD4^CreErt2/wt^ × Ai14 pulse-chase assays. **a**, Schematic representation of the CD4^CreErt2/wt^ × Ai14^flox/flox^ timed mating strategy. **b**,**c**, Confocal images of E14.5 embryos from CD4^CreErt2/wt^ × Ai14^flox/flox^ timed matings, highlighting the presence of tdTom^+^ macrophages within embryos from plugged females pulsed with a single dose of 4-OHT and progesterone at E7.5 (**b**) or E8.5 (**c**). Images are representative of five embryos from three timed mating experiments. **d**, Confocal images of the brain highlighting several clusters of tdTom^+^ perivascular macrophages within the meninges of postnatal day 14 pups (left image). Images are representative of three mice obtained from two timed mating experiments. **e**, Images highlighting the presence of CD4-lineage tdTom^+^F4/80^+^ macrophages within the liver. Images are representative of four mice from three timed mating experiments. **f**, Confocal image of the liver highlighting the expression of Clec4F on tdTom^+^ macrophages, identifying them as Kupffer cells. The image is representative of two mice from a single timed mating experiment. Icons in **a** created in BioRender; Jain, R. https://biorender.com/f6aluj6 (2026).

To directly determine whether early CD4-expressing embryonic progenitors contribute to macrophage development later in ontogeny, we used an inducible lineage-tracing approach. CD4 CreErt2 knock-in male mice^59^ were paired with Ai14 female mice^57^ for timed mating (Fig. 7a), and a single pulse of 4-hydroxytamoxifen (4-OHT) was administered at E7.5, E8.5 or E9.5 to label CD4-expressing cells present within these discrete developmental windows. Embryos were subsequently collected at E14.5 for sectioning and immunostaining.

**Extended Data Fig. 7:**
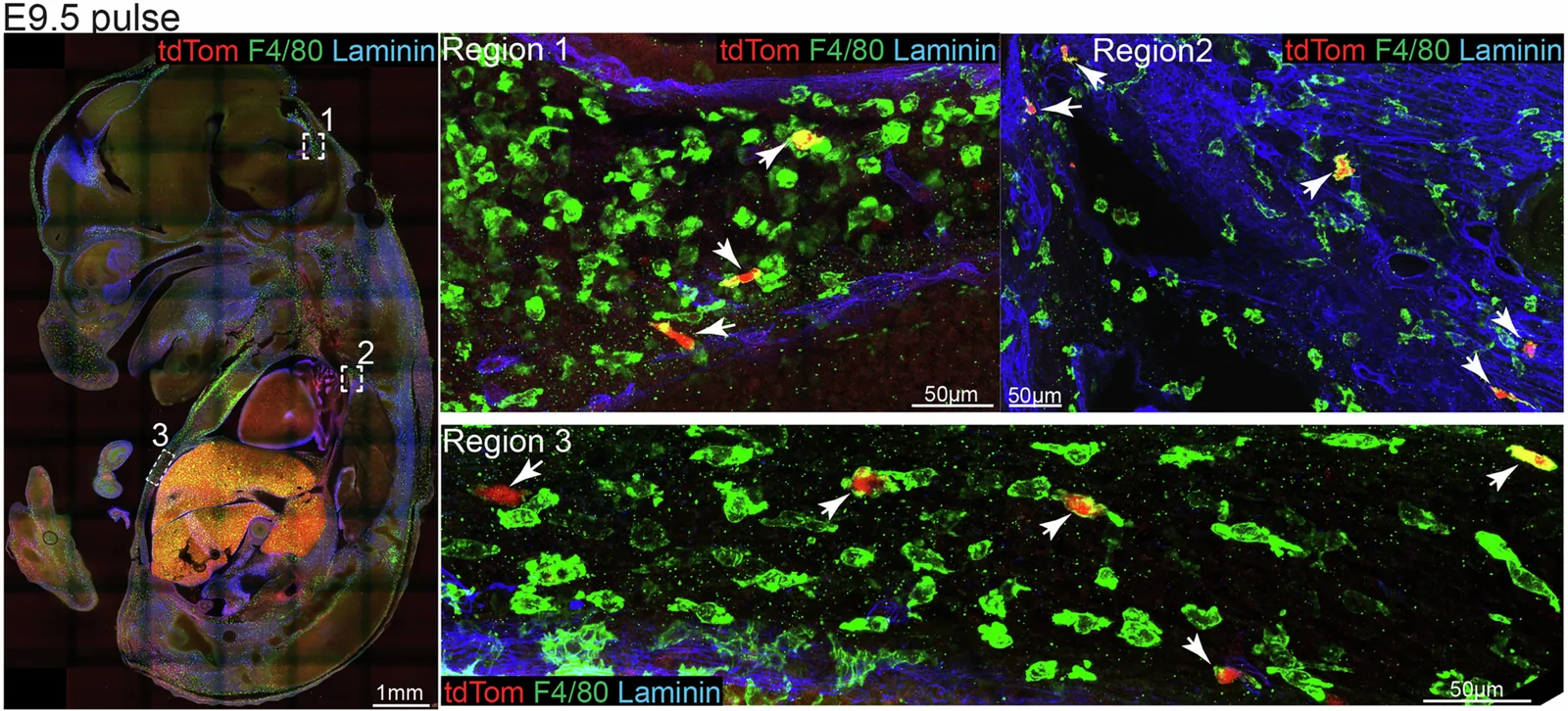
Cd4^CreErt2/wt^ x Ai14 pulse chase assays. Confocal microscopy of E14.5 embryos from Cd4^CreErt2/wt^ x Ai14 timed matings, highlighting the presence of tdTom+ macrophages within embryos pulsed with a single dose of 4^−^OH-Tamoxifen and Progesterone at E9.5. Data representative of 6 embryos from 4 timed mating experiments.

Confocal analysis revealed numerous tdTom^+^F4/80^+^ macrophages throughout the embryo following induction at all three time points, with particularly prominent labeling observed in the developing meninges and lower embryonic body regions (Fig. 7a–c and Extended Data Fig. 7). The contribution was most pronounced following E7.5 and E8.5 induction, indicating that CD4-expressing progenitors arising during the earliest phases of embryogenesis generate a substantial fraction of fetal macrophages detectable at E14.5. These findings provide direct temporal fate-mapping evidence that early CD4^+^ embryonic progenitors, consistent with the developmental window of eEMPROR emergence, contribute to downstream macrophage populations in vivo.

To determine whether early CD4-marked embryonic progenitors also generate macrophage populations that persist after birth, we performed an additional inducible lineage-tracing experiment, administering a single pulse of 4-OHT at E8.5. Pups were delivered by cesarean section, fostered by Swiss mothers and analyzed on postnatal day 14. Brain and liver tissues were dissected, sectioned and examined by confocal microscopy. Consistent with our embryonic tracing studies, tdTom^+^ CD4-lineage macrophages remained readily detectable within the meningeal compartment (Fig. 7d) as well as in the liver (Fig. 7e,f). These findings demonstrate that CD4-expressing progenitors labeled during this early developmental window give rise not only to fetal macrophages but also to tissue-resident macrophage populations that persist into postnatal life.

### CD4^+^ erythrocytic cells in early human placental tissue

**Fig. 8:**
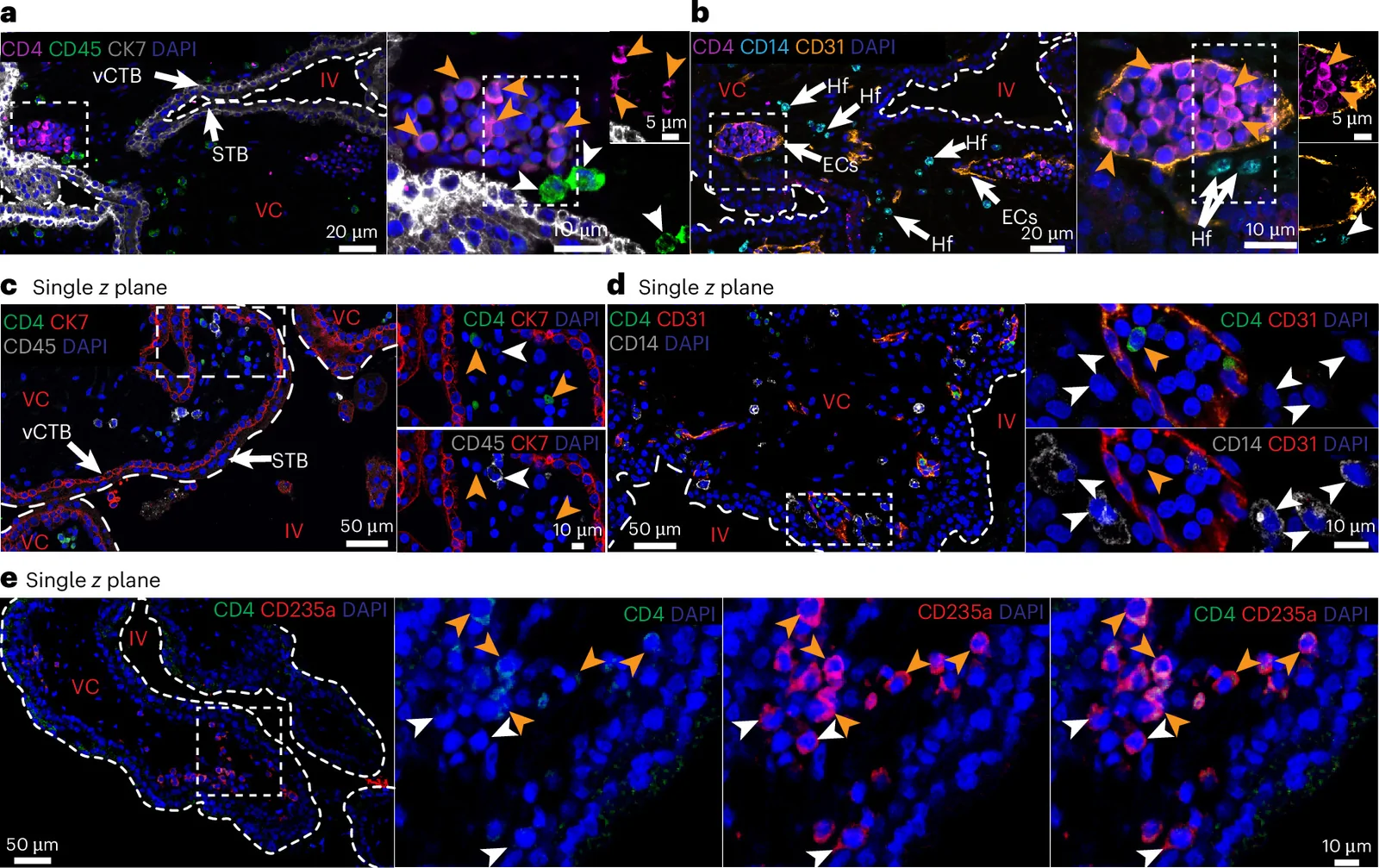
Identification of CD4^+^ cells at the maternal–embryonic interface in human placental tissue. **a**,**b**, Images of first-trimester placental tissues obtained at 5 weeks and 1 day of gestation, highlighting the presence of CD4^+^ cells within the embryonic villi. **a**, Costaining of the tissue with CD4, CD45, CK7 and DAPI, highlighting the presence of CD4^+^ cells (orange arrowheads) in a cell cluster (DAPI staining) within the villous core. White arrowheads indicate the mutual exclusion of CD4 and CD45 staining. **b**, Costaining of serial sections with CD4, CD14, CD31 and DAPI, highlighting the presence of clusters containing CD4^+^ cells (orange arrowheads) encapsulated by CD31-expressing cells. Hofbauer cells within the villous core were identified by the expression of CD14 (white arrowhead), and these cells did not show CD4 expression (single-color insets). **c**, Confocal images of first-trimester placental and decidual tissues obtained at 5 weeks and 2 days of gestation, highlighting the localization of CD4^+^ cells within the villous core (marked by CK7 staining). These CD4^+^ cells (orange arrowheads) did not show CD45 expression (white arrowheads). **d**, Confocal images highlighting the localization of CD4^+^ cell regions demarcated by CD31^+^ cells. These CD4^+^ cells (orange arrowheads) did not show CD14 expression (white arrowheads). **e**, Confocal images highlighting the localization of CD4^+^ cells relative to embryonic erythrocytes, identified as nucleated CD235a^+^ cells. Both CD4^+^CD235a^+^ (orange arrowheads) and CD4^−^CD235a^+^ (white arrowheads) erythrocytes can be visualized within the tissue section. Images in **a** and **b** are representative of six individual placental tissues. In **c**–**e**, the images are representative of two placental tissues. VC, villous core; IV, intervillous space; vCTB, villous cytotrophoblasts; STB, syncytiotrophoblasts; Hf, Hofbauer cells; ECs, endothelial cells.

Given the parallels between human and mouse embryonic hematopoiesis, we next asked whether CD4^+^ erythro-myeloid lineages could be detected in early human extraembryonic tissues before HSC emergence^60^. We analyzed early first-trimester placental tissue from pregnancies at 5 weeks of gestation, corresponding developmentally to approximately a 3-week-old human embryo. Serial sections were stained with antibodies to CD4, CD45, CD14, CD31, cytokeratin 7 (CK7) and DAPI. The villous core was identified by its characteristic architecture: a mesenchymal compartment covered by villous cytotrophoblasts and an overlying syncytiotrophoblast layer, which together separate it from the surrounding intervillous space^61^ (Fig. 8a). Immunostaining revealed numerous CD45^+^ leukocytes dispersed throughout the villous core, whereas CD4^+^ cells were confined to discrete cellular aggregates (Fig. 8a). In serial sections stained for CD4, CD31, CD14 and DAPI, these CD4^+^ cells were found exclusively within CD31^+^ endothelial-lined structures, indicating their intravascular localization (Fig. 8b). CD14^+^ Hofbauer cells^62^ were readily detected within the villous stroma; however, none of the CD14^+^ cells coexpressed CD4 (Fig. 8b). These findings suggest that, similar to the mouse eEMPROR cell lineage, early human CD4^+^ erythro-myeloid cells are present within nascent villous vasculature, whereas Hofbauer cells lack CD4 expression.

**Extended Data Fig. 8:**
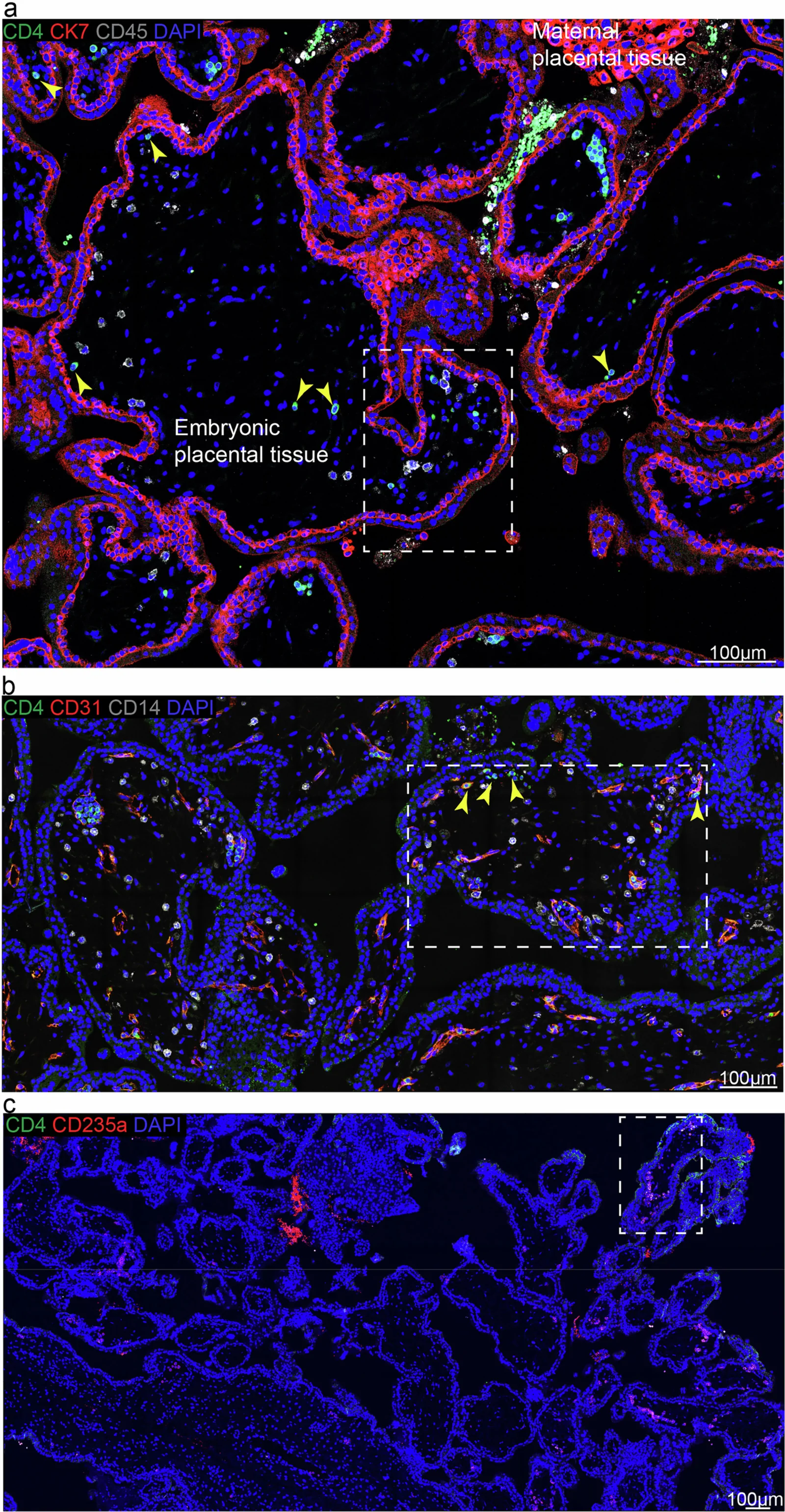
Identification of CD4^+^ cells at the maternal-embryonic interface in human placental tissue. Confocal imaging of large fields of view highlighting the presence of CD4^+^ cells within the embryonic villi visualized within first-trimester placental and decidual tissues from a 5-week 2-day gestation (see Fig. 8 for more details). **a**, Image highlights the localization of CD4^+^ cells within the villous core (marked by CK7 staining). These CD4^+^ cells (green, highlighted by yellow arrowheads) did not show expression of CD45 (white); **b**, Confocal image highlights the localization of CD4^+^ cell regions demarcated by CD31^+^ cells (red). These CD4^+^ cells (green, highlighted by yellow arrowheads) did not show expression of CD14 (white); **c**, Confocal image highlighting the localization of CD4^+^ cells (green) in context of embryonic erythrocytes as identified by nucleated CD235a^+^ cells (red). Both CD4^+^CD235a^+^ and CD4^−^CD235a^+^ erythrocytes can be visualized within the tissue section. **a-c**, data representative of 2 placental tissues.

To extend these observations across a broader anatomical context, we performed confocal imaging of immunostained sections from human placental tissues obtained at 5 weeks and 2 days of gestation, enabling visualization of large regions of the maternal–embryonic interface and multiple villous units (Extended Data Fig. 8). Consistent with our earlier findings, CD4^+^ cells were detected within discrete intravascular clusters across numerous embryonic villi (Fig. 8c,d and Extended Data Fig. 8a,b). These clusters were encased by CD31^+^ endothelial cells; however, the CD4^+^ cells themselves lacked expression of CD45 (Fig. 8c) and CD14 (Fig. 8d and Extended Data Fig. 8b), indicating that they do not correspond to leukocytes or Hofbauer cells. To further define their identity, we stained additional sections with CoraLite Plus 488-conjugated anti-CD4 and CoraLite 594-conjugated anti-CD235a antibodies. This revealed both CD4^+^CD235a^+^ and CD4^−^CD235a^+^ nucleated erythrocytes within villous vessels (Fig. 8e and Extended Data Fig. 8c). The presence of nucleated erythroid cells confirms that active erythropoiesis is occurring within early human villi, and the identification of a subset of CD4^+^ erythroid cells suggests the existence of a CD4-marked precursor population. This putative precursor population mirrors the eEMPROR lineage identified in mouse MIXER and appears well before the emergence of functional fetal HSCs, which later give rise to CD4-expressing hematopoietic lineages.

## Discussion

Our work identifies the maternal–fetal interface as a previously unrecognized extraembryonic hematopoietic territory active during the earliest phases of erythro-myeloid development. Within this transient MIXER tissue, we detected not only classical CSF1R^+^ EMPs but also a CD4^+^ EMP population—termed eEMPROR cells—with broad lineage potential and sustained contributions to fetal and postnatal macrophage compartments. These findings substantially broaden the prevailing yolk sac-centered model of primitive hematopoiesis and suggest instead that the earliest EMP waves arise across anatomically distinct extraembryonic niches.

The transient nature and anatomical inaccessibility of MIXER likely explain why this compartment has remained largely undetected in previous studies. Unlike the yolk sac, which can be readily isolated as a discrete membranous tissue during embryo collection, the maternal–fetal interface forms a minute implantation-associated tissue tightly apposed to decidual and trophoblastic structures and is therefore routinely excluded from standard embryonic preparations. In addition, unequivocal recognition of embryonic hematopoietic cells at this site requires genetic lineage discrimination; without such labeling, cells at the implantation boundary would likely be interpreted as maternally derived. Conventional flow cytometric strategies may further obscure this compartment by excluding CD4^+^ cells during lineage depletion. Thus, MIXER has remained positioned at the intersection of anatomical, technical and analytical blind spots in early hematopoietic studies.

A central question raised by our findings is whether MIXER merely represents a passive reservoir that transiently accumulates yolk sac-derived progenitors. Several features strongly argue against this interpretation. Hematopoietic activity within the MIXER region is already evident at E7.5, contemporaneous with the first hematopoietic wave in the yolk sac. By E8.5, however, the maternal–fetal interface contains not only abundant CSF1R^+^ EMPs but also a selective enrichment of eEMPROR cells, which are absent from the corresponding yolk sac preparations. If MIXER simply receives progenitors from the yolk sac, one would expect its hematopoietic composition to largely mirror that of the yolk sac. Instead, the maternal–fetal interface displays both a distinct progenitor abundance and a progenitor composition that is not readily explained by passive redistribution. This conclusion is reinforced by the presence of embryonic endothelial structures marked by CD31 and CD309, along with the broad tissue-wide distribution of embryonic erythroid and leukocytic cells before the establishment of robust embryonic–extraembryonic circulation. More broadly, the developmental hallmarks that define the yolk sac as an embryonic hematopoietic territory, including early progenitor enrichment, association with hemogenic endothelial structures, multilineage hematopoietic output and downstream lineage contribution, are likewise exhibited by MIXER, making it highly implausible that MIXER functions solely as a passive reservoir at the maternal–fetal interface. Nevertheless, limited progenitor movement between contiguous extraembryonic compartments cannot be fully excluded.

The embryological positioning of MIXER further suggests that its contribution is biologically purposeful rather than incidental. Whereas the yolk sac is associated with the vitelline extraembryonic vascular axis, MIXER is located at the maternal-facing chorionic implantation interface, which develops in continuity with the placental territory. Beginning at approximately E8.5, these regions become connected to the embryo through two spatially distinct extraembryonic vascular beds, the vitelline circulation and the emerging placental-side vascular network. Thus, even as embryonic perfusion becomes established, the yolk sac and MIXER remain situated within distinct endothelial, trophoblastic, metabolic and maternal contexts. This spatial compartmentalization raises the possibility that the earliest EMPs are generated across physiologically specialized niches rather than within a single uniform extraembryonic source. One plausible model is that mesodermal progenitors emerging from the primitive streak seed both the yolk sac and MIXER, where distinct local microenvironments subsequently shape divergent hematopoietic programs.

Within this framework, the preferential association of eEMPROR cells with MIXER is particularly notable. In contrast to canonical CSF1R^+^ EMPs, these CD4^+^ progenitors display a distinct phenotypic profile, broad erythro-myeloid differentiation capacity, and sustained contribution to fetal and postnatal macrophage populations, with particularly prominent representation in PVM niches. Whether these properties reflect functional specialization or intrinsic developmental programming, or are imposed by local stromal and endothelial signals at the maternal–fetal boundary remains unresolved. Defining how trophoblast-associated cues, hemogenic endothelium, growth factor availability and mechanical forces shape progenitor fate at this earliest interface will be important for understanding how microenvironmental diversity is translated into myeloid lineage diversification before circulation begins. Future single-cell transcriptomic, spatial and epigenomic approaches should help resolve the cellular architecture of MIXER, clarify its developmental relationship to the yolk sac, and identify the stromal, endothelial and trophoblast-associated signaling programs that establish its unique hematopoietic identity.

Human studies have identified placental EMPs as early, HSC-independent progenitors that arise within first-trimester villi and generate Hofbauer cells before embryo–placenta circulation is established^34,35,63^. This timing is consistent with the fact that vitelline–embryonic–placental perfusion develops later in gestation^34,64^. In line with this early window, our 5-week human villous analyses reveal the presence of CD4^+^CD235a^+^ nucleated erythroid-like cells within CD31^+^ endothelial-lined structures. However, CD14^+^ Hofbauer cells do not show expression of CD4, suggesting that, paralleling mouse eEMPROR cells, an as-yet undeciphered CD4-lineage EMP might exist within early placental tissue. As both placental EMPs and CD4^+^ erythroid-like cells appear before placental perfusion, their presence indicates in situ hematopoiesis at the human maternal–fetal interface. These findings suggest that, similar to mice, the early human placenta may support multiple erythro-myeloid programs.

How MIXER relates to the later placenta, which itself acquires hematopoietic activity during gestation, remains unresolved. One possibility is that MIXER represents a transient preplacental interface that supplies progenitors before placental perfusion is fully established. Such a niche would be uniquely exposed to perturbations in trophoblast integrity, decidual health, uterine blood flow and maternal inflammation, raising the possibility that disruption of this earliest maternal–fetal hematopoietic environment could have lasting consequences for macrophage ontogeny and developmental immune homeostasis.

Taken together, our findings broaden both the spatial and temporal frameworks of mammalian embryonic hematopoiesis. Rather than arising exclusively from a yolk sac-dominated program, the earliest EMP waves appear to involve coordinated contributions from anatomically distinct extraembryonic territories. Defining how MIXER-derived progenitors integrate with yolk sac- and embryo-derived precursor populations, and whether they perform specialized developmental functions not captured by current models, is important for understanding how the earliest blood and immune lineages are positioned to support organogenesis and long-term tissue homeostasis.

## Methods

### Human tissues

First-trimester placental and decidual tissues were obtained from women choosing elective nonmedical pregnancy termination. Free and fully informed consent was obtained from the women choosing these procedures. The collection and use of human samples were approved by the Ethics Committee of the Medical University of Vienna (approval no. EK2515-2024), with ethical approval renewed annually. All experimentation was conducted in accordance with the Declaration of Helsinki and Austrian laws and guidelines. All safety-related and regulatory aspects of the project were carried out in accordance with the stringent guidelines outlined by the Medical University of Vienna and Vienna General Hospital. All the samples that were imaged were destroyed after analysis. No compensation was provided to the participants.

### Immunofluorescence staining of first-trimester human placental tissue

First-trimester placental tissues were fixed in 7.5% formaldehyde and subsequently embedded in paraffin. Tissue sections with a thickness of 2.5 μm were prepared using an HM355 microtome (Microm). The sections were deparaffinized by incubating in xylene for 10 min, followed by a graded rehydration series using 100%, 96% and 70% ethanol, and then rinsed in deionized water. Antigen retrieval was carried out in citrate buffer (pH 6.0) using a KOS Microwave Station (Milestone).

For immunofluorescence staining, sections were blocked for 1 h in 1× TBS/T containing 5% normal goat serum. Primary antibodies to the following markers were applied, followed by incubation overnight at 4 °C: CD14 (clone D7A2T, 75181S, Cell Signaling, dilution 1:500), CD4 (clone CL0395, AMAB90754, Sigma, dilution 1:100), CD45 (clone D9M8I, 13917S, Cell Signaling, dilution 1:200) and CK7 (clone OV-TL 12/30, M7018, Dako, dilution 1:200) (Supplementary Table 1). Following three washing steps with TBS/T, the sections were incubated for 1 h at 22 °C with DAPI (final concentration 1 µg ml^−1^) and the following secondary antibodies: anti-mouse IgG Alexa Fluor 488 (A-11017, Thermo Fisher Scientific, final concentration 2 µg ml^−1^), anti-rabbit IgG Alexa Fluor 488 (A-11070, Thermo Fisher Scientific, final concentration 2 µg ml^−1^), anti-mouse IgG Alexa Fluor 568 (A-11019, Thermo Fisher Scientific, final concentration 2 µg ml^−1^) and anti-rabbit IgG Alexa Fluor 647 (A-21245, Thermo Fisher Scientific, final concentration 2 µg ml^−1^). Afterward, sections were washed three times in TBS/T and stained for 1 h at 22 °C with CD235a-PE (clone HI264, 349105, BioLegend, final concentration 2 µg ml^−1^) and CD31-PE (clone REA1312, 130-128-769, Miltenyi, dilution 1:100). For immunofluorescence co-staining with directly labeled antibodies, antigen retrieval of deparaffinized sections was carried out in Tris-EDTA buffer (pH 9.0) using a KOS Microwave Station (Milestone SRL). The sections were blocked for 1 hour in PBS containing 3% BSA. We applied directly labeled antibodies, CD235a-594 (Clone 2B12G10, #CL594-66778, Proteintech, dilution 1:200), CD4-488 (Clone 240427E12, #CL488-83513-7, Proteintech, dilution 1:200), and DAPI (final concentration 1 µg/mL) in PBS containing 1% BSA and incubated for 2 hours at room temperature. The samples were washed extensively and mounted on glass slides for imaging. Imaging was performed using a fluorescence microscope (Olympus BX50) equipped with a Hamamatsu ORCA-Spark digital camera, and images were analyzed using Olympus cellSens Standard software. Confocal imaging for some of these experiments was performed using a Leica SP8 confocal microscope.

### Mice

Transgenic mice were bred and maintained within the Animal Facility at the Centenary Institute of Cancer Medicine and Cell Biology; the Core Facility Laboratory Breeding and Husbandry (CFL, Himberg site), Vienna; and the Core Facility Laboratory Breeding and Husbandry (CFL, AKH site), Medical University of Vienna/AKH, Vienna, Austria. Mice were maintained under specific pathogen-free conditions at 22–26 °C with a 12-h light/dark cycle and relative humidity of 55 ± 10%. C57BL/6J and C57BL/6JRj mice were obtained from commercial vendors, namely the Australian BioResources facility in Moss Vale, New South Wales, Australia; the Animal Resource Centre in Canning Vale, Western Australia, Australia; and Janvier Labs, France. mT/mG mice^43^ were purchased from The Jackson Laboratory. DPE-GFP mice^47^ (also detailed in the accompanying article^41^), β-actin-Cre mice^42^, CD4-Cre mice^58^, Ai14 mice^57^ and CD4-CreErt2 mice^59^ (*Cd4*^*tm1(cre/ERT2)Thbu*^) were used as detailed throughout the article. Unless otherwise specified, mice of either sex aged between 6 and 24 weeks were used for experimentation. All experiments were conducted in accordance with the ethics protocols approved by the Animal Ethics Committee of the Sydney Local Health District, the Animal Ethics Committee of the University of Sydney, and the Bundesministerium für Frauen, Wissenschaft und Forschung (Federal Ministry for Women, Science and Research) of the Republic of Austria. All experiments were conducted using approved standard operating procedures and flow cytometry biosafety protocols. Mice were killed by CO_2_ asphyxiation or cervical dislocation. For embryo collection, pregnant female mice were killed, and the embryos were surgically excised and exsanguinated. Individual strains used in this study are listed throughout the article.

### DPE-GFP mouse strain

The DPE-GFP mouse strain was generated by microinjecting a construct containing the distal and proximal CD4 enhancer elements, based on the pTG construct originally described by Siu and colleagues^65^. In DPE-GFP mice, GFP is expressed in all T cells, including naive and effector populations^47,66^. We subsequently discovered that GFP expression is not restricted to T cells; plasmacytoid dendritic cells^50^, PVMs^48^ and some progenitor populations such as EMPROR cells^41^ also exhibit robust GFP expression in these mice.

### Timed mating

For timed mating experiments, female and male mice were paired according to the experimental requirements. Briefly, 2–3 days before the initiation of timed mating, soiled bedding from cages housing male mice was transferred to the cages of female mice to promote synchronization of estrous cycles (Whitten effect). On the day of timed matings, female mice were paired with the respective males and allowed to mate overnight. Thereafter, the female mice were checked each morning for vaginal plugs. Those with vaginal plugs were immediately separated and used as needed for the experiments.

### Tamoxifen treatment

Mice used for timed mating were treated as described previously^14^. Briefly, a single dose of 4-hydroxytamoxifen (75 µg g^−1^) supplemented with 37.5 μg g^−1^ progesterone was administered to pregnant females at E7.5, E8.5 or E9.5 depending on the experimental plan. Embryos were collected at E14.5, and a small portion of tail or forelimb tissue was dissected for PCR genotyping. Additionally, tissues were collected from P14 pups according to the experimental requirements.

### Tissue dissection

Pregnant mice were selected at specific time points and euthanized. Embryos were then collected for processing, imaging and flow cytometry. Microdissections were performed using an Olympus SZ51 stereomicroscope equipped with an Olympus KL1600 LED light source. Embryos were staged based on the presence of developmental landmarks according to Theiler stages^44^ and Downs and Davies^67^ to exclude outliers. The microdissection strategy is outlined in Extended Data Fig. 1. Briefly, individual conceptuses were isolated from the gravid uterus with care taken to prevent rupture. For confocal imaging, the separated conceptuses were fixed with 4% paraformaldehyde (PFA) overnight at 4 °C and then processed for imaging as detailed below.

For flow cytometry, the tissue was further microdissected. Briefly, the myometrium was removed, and the conceptus was split open at one end to release the embryo encased in the yolk sac. The maternal tissue was further microdissected to isolate the MIXER tissue. E8.5 embryos and yolk sacs were separated, and all stage-matched tissues were pooled into their respective sample tubes. E8.5 embryos were staged based on the fusion of the headfold, the number of somite pairs, the turning of the embryo, and the development, localization and absence of fusion of the allantois with the chorion. We did not observe rhythmic or continuous heartbeats during embryo collection. The tissues were processed for flow cytometry as detailed below. During microdissection, the allantois was included in the embryo sample (as it originates from the embryo), and the chorion was included in the yolk sac.

### Macroscopic and microscopic evaluation of dissected tissues

For macroscopic evaluation, the tissues were dissected as described above. All tissues were fixed overnight with 4% PFA, washed once with 1× PBS and mounted in a Petri dish for imaging using a Zeiss Lumar V12 stereomicroscope at the Core Imaging Facility, Anna Spiegel Forschungsgebäude. Additionally, the tissues were remounted on slides and imaged using an Olympus laser scanning confocal microscope (FV3000) equipped with 405-, 488-, 561- and 640-nm OBIS diode lasers (Coherent). All data were analyzed using Fiji/ImageJ^68,69^ (https://imagej.net/ij).

### Confocal imaging

Confocal laser scanning microscopy was performed on tissue sections or whole mounts according to previously established protocols^48^. Briefly, tissues of interest were dissected and fixed overnight with 4% PFA at 4 °C. Following fixation, all tissue samples were washed with immunostaining buffer (1× PBS, 2% FCS, 2 mM EDTA, 0.2% azide, 0.3% Triton X-100). In the case of whole-mount preparations (for example, E8.5 embryos and maternal conceptuses), the intact tissues were washed with immunostaining buffer and processed for confocal microscopy. Other tissues, such as the gravid uterus (Fig. 1c) and E14.5 embryos (Extended Data Fig. 6b), were embedded in 5% low-melting agarose and sectioned into 100- to 200-μm-thick serial sections using the Vibratome 1000 Plus sectioning system. Tissues were stained with primary antibodies (Supplementary Table 1) in immunostaining buffer for 48–72 h on a rocker at 4 °C. After staining, the samples were washed three times with immunostaining buffer at 22 °C, with each wash lasting a minimum of 30 min. Tissues were then stained with secondary antibodies (Supplementary Table 1) in immunostaining buffer overnight on a rocker at 4 °C. Thereafter, the samples were washed three times with immunostaining buffer at 22 °C, for a minimum of 30 min per wash. Tissues were mounted on clean slides using DABCO and imaged with a Leica TCS SP5 confocal laser scanning microscope system equipped with an acousto-optical beam splitter and hybrid detectors for sensitive signal detection, or with an Olympus confocal microscope (detailed in the section above). For pulse-chase assays, imaging was performed using an Olympus spinning disc confocal microscope. Image analysis was carried out using Volocity software (PerkinElmer) and Fiji/ImageJ^68,69^ (https://imagej.net/ij).

### Cryosectioning

Tissues of interest were embedded in optimal cutting temperature compound and cryosectioned at a thickness of 15 μm. Sections were fixed in 4% PFA for 30 min at 22 °C and washed three times with 1× PBS. Sections were then blocked with 10% goat serum in immunostaining buffer for 30 min at 22 °C, followed by incubation with primary antibodies (Supplementary Table 1) overnight at 4 °C. Sections were washed three times with immunostaining buffer and once with PBS, then incubated with secondary antibodies (Supplementary Table 1) for 3 h at 22 °C in the dark. Sections were washed three times with immunostaining buffer, stained with DAPI for 5 min at 22 °C, washed again and mounted with mounting medium. Slides were coverslipped and stored at 4 °C in the dark until imaging.

### Embryonic tissue dissection and processing for flow cytometric analysis

Flow cytometry antibodies are detailed in Supplementary Table 2, while the panels used are described in the individual figures. At E8.5, yolk sac, embryo proper and MIXER tissues were collected into 1× PBS containing 10% FBS (Fig. 1a and Extended Data Fig. 1). The samples were subsequently digested with 0.2 mg ml^−1^ collagenase type IV (Sigma, C5138-1G) for 20–30 min at 37 °C with intermittent mixing. They were gently passed through an 18- or 19-gauge needle to obtain a homogeneous sample. The cells were centrifuged, washed with FACS wash and transferred to a 96-well U-bottom plate before staining for flow cytometry.

For E14.5 embryos, organs were dissected and processed as described below.

#### Liver

Livers were dissected, finely chopped in 1× PBS and passed through a 19-gauge needle to obtain a homogeneous cell suspension. Cells were filtered through a 100-μm cell strainer and centrifuged at 754*g* for 10 min at 4 °C. Samples were treated with ACK lysing buffer to lyse RBCs for 5–10 min at 37 °C, neutralized with FACS wash, centrifuged, and resuspended in FACS wash before being transferred to a 96-well U-bottom plate for staining and subsequent flow cytometry analysis.

#### Blood

Blood from embryos was collected into Alsever’s solution (0.4% w/v sodium chloride (Sigma, cat. no. 310166), 0.8% w/v sodium citrate (Sigma, cat. no. S1804), 0.05% w/v citric acid monohydrate (Chem-Supply, cat. no. CA014), 2% w/v d-glucose (Sigma, cat. no. G8270) in deionized water, pH 7.3). The blood cell suspension was washed in FACS wash, centrifuged at 754*g* for 10 min at 4 °C, subjected to RBC lysis using ACK lysing buffer for 10 min at 37 °C, and subsequently neutralized with FACS wash and centrifuged at 524*g* for 5 min at 4 °C. The supernatant was discarded, and the cell pellet was resuspended in FACS wash before being transferred to a 96-well U-bottom plate for staining and subsequent flow cytometry.

#### All other organs

All other organs were finely chopped into small pieces in 1× PBS containing 10% FBS, 0.2 mg ml^−1^ collagenase type IV and 2% v/v DNase (Sigma, cat. no. D5025-150KU), then passed through a 19-gauge needle to obtain a homogeneous cell suspension. Cells were incubated for 30 min at 37 °C. Cells were then filtered through a 100-μm cell strainer and centrifuged at 754*g* for 10 min at 4 °C. Samples were subjected to RBC lysis using ACK lysing buffer for 5–10 min at 37 °C, neutralized with FACS wash, centrifuged at 754*g* for 10 min at 4 °C, and resuspended in FACS wash before being transferred to a 96-well U-bottom plate for staining and subsequent flow cytometry.

### Sorting of embryonic precursor populations

Different embryonic tissues, including MIXER, yolk sac and embryo proper tissues, were microdissected and processed. Briefly, E8.5 embryo proper, yolk sac and MIXER tissues were collected into 1× PBS containing 10% FBS. Afterward, they were digested using 0.2 mg ml^−1^ collagenase type IV (Sigma, C5138-1G) for 20–30 min at 37 °C with intermittent mixing. Next, they were gently passed through an 18- or 19-gauge needle to obtain a homogeneous sample. Cells were subsequently centrifuged, washed in sorting buffer (1× HBSS (Gibco, cat. no. 14175-095) + 2% FBS (HyClone, cat. no. SH30084.03) + 0.2% EDTA), filtered through a 70-μm cell strainer and transferred to a 96-well U-bottom plate for staining before sorting. Cells were sorted using the BD FACSAria II cell sorter. Sorted cells were collected into 5-ml polypropylene tubes containing 100–200 μl of 100% FBS (double filtered using 0.45-μm filters). The post-sort purity of each population was evaluated. Only sorted populations with >95% purity were used for experiments.

#### Sorting of precursors from E14.5 livers

Livers were dissected from E14.5 embryos and processed as described above. Cell populations were sorted into 5-ml polypropylene tubes containing 100–200 μl of 100% FBS using the BD FACSAria II cell sorter. The post-sort purity of each population was evaluated, and samples with >95% purity were used for experiments.

### Erythro-myeloid potential of the embryonic precursor–OP9 system

A few days before coculture, OP9 cells were plated in six-well plates (Corning, cat. no. 430167) to achieve 80–90% confluence by the day coculture was initiated. OP9 cells were maintained in MEM (Sigma, cat. no. M8042) containing 20% FBS, 2% penicillin–streptomycin (Gibco, cat. no. 15140-122), 1% L-glutamine (Gibco, cat. no. 25030-081) and 1× tissue-culture grade β-mercaptoethanol (Gibco, cat. no. 21985-023). On the day of coculture, MIXER and yolk sac tissues were dissected from the embryos. The tissues were processed and sorted as described above. Sorted cells were seeded onto 80–90% confluent OP9 stromal cell layers in medium supplemented with SCF, IL-3, IL-6, EPO, TPO, GM-CSF and M-CSF. Plates were routinely monitored, and after hematopoietic colonies were visualized, they were flushed with 1× HBSS + 10% FCS, washed, stained, and analyzed for EMP potential using flow cytometry (BD LSR Fortessa) and cytology.

### Erythro-myeloid potential of embryonic precursors

Fetal liver cells were isolated, processed and sorted as described above. For determining erythro-myeloid potential, duplicate samples containing 500 sorted cells were suspended in 1.5 ml MethoCult GF M3434 (STEMCELL Technologies, cat. no. 03434) with SCF, IL-3, IL-6 and EPO and further supplemented with 5 ng ml^−1^ TPO (Prospec, cat. no. CYT-346), 3 ng ml^−1^ GM-CSF (Prospec, cat. no. CYT-222) and 5 ng ml^−1^ M-CSF (PeproTech, cat. no. 0315-02-10). The cell suspension was plated in two 35-mm Petri dishes and incubated at 37 °C. Two weeks later, individual colonies were randomly selected. We obtained 20–30 distinct colonies from each of the eEMPROR and cMoP populations. In each experiment, approximately 20–25 colonies per group were analyzed using flow cytometry, while 5–10 colonies per group were used for Giemsa staining and cytological evaluation. For monocytes that formed clusters of three to four cells that could not be assessed individually, the entire contents of the MethoCult plate were collected and analyzed by flow cytometry, Giemsa staining and cytology. All samples were washed twice in FACS wash (1× PBS, 2% FBS, 2 mM EDTA, 0.01% azide) before being transferred to a 96-well U-bottom plate for staining and flow cytometric determination of erythro-myeloid potential.

### Giemsa staining and cell cytology

Giemsa staining was performed according to the previously established protocol^70^. Briefly, colonies from cultures were isolated and resuspended in 200 μl FACS buffer in a 96-well U-bottom plate. The plate was centrifuged at 931*g* for 5 min at 22 °C. The cell pellet was washed twice to remove traces of medium or MethoCult. The cell pellet was resuspended in 100 μl of 1× HBSS, and the sorted cells were centrifuged onto glass slides for 5 min at 500 rpm (28.23*g*) using a Cytospin 3 cytocentrifuge (Shandon). The slides were air-dried overnight, fixed with methanol for 5 min, air-dried again and stained for 30 min with Giemsa (Sigma-Aldrich, cat. no. GS1L-1L). The slides were gently washed with tap water to remove excess stain, dried at 22 °C and then imaged. Imaging was performed using the Leica DM6000B microscope with a 20× objective lens. A tiled mosaic of the entire region containing stained cells was acquired and analyzed for the presence of specific cell lineages.

### Cell lines used in this study

OP9 cells used in this study were a kind gift from S. Naik (Walter and Eliza Hall Institute of Medical Research). These cell lines are not listed in the International Cell Line Authentication Committee Register of Misidentified Cell Lines.

### Statistical analysis

Prism software (GraphPad Software) was used for statistical analysis. Statistical tests were used as described in the figure legends. Statistical significance was assumed when *P* < 0.05. No statistical methods were used to predetermine sample sizes, but our sample sizes are similar to those reported in previous publications. Data distribution was assumed to be normal, but this was not formally tested. The experiments were not randomized. Data collection and analysis were not performed blind to the conditions of the experiments.

### Reporting summary

Further information on research design is available in the Nature Portfolio Reporting Summary linked to this article.

## Online content

Any methods, additional references, Nature Portfolio reporting summaries, source data, extended data, supplementary information, acknowledgements, peer review information; details of author contributions and competing interests; and statements of data and code availability are available at https://doi.org/10.1038/s41590-026-02683-4.

## Supplementary information

Supplementary Information Supplementary Tables 1 and 2.

Reporting Summary

## Source data

Source Data Fig. 4 Statistical source data.

Source Data Fig. 6 Statistical source data.

Source Data Extended Data Fig. 2 Statistical source data.

## Data Availability

Datasets generated in this study will be available on figshare (https://doi.org/10.6084/m9.figshare.34031043)^71^. Source data are provided with this paper.

## Code availability

No code was used in this study.
