# Supplementary material for: The maternal–fetal interface is an early hematopoietic niche enriched in distinct erythro-myeloid progenitors
Source: Nat Immunol. 2026 Oct 26;27(11):2306–21. doi: 10.1038/s41590-026-02683-4 (PMC13710174; doi:10.1038/s41590-026-02683-4)
Supplement: Supplementary file 1 [file 41590_2026_2683_MOESM1_ESM.pdf]

nature immunology

Supplementary information https://doi.org/10.1038/s41590-026-02683-4

The maternal–fetal interface is an early hematopoietic niche enriched in distinct erythro-myeloid progenitors

In the format provided by the authors and unedited

NI-A41989A_ Supplementary Tables 1 and 2

Supplementary Table 1: Antibodies for immunostaining

**Antibodies for human tissues**	**Clone name/Isotype**	**Dilution**	**Vendor**	**Cat. Nr.**
Anti-CD14	D7A2T/Rabbit IgG	1:500	Cell Signaling	75181S
Anti-CD4	CL0395/Mouse IgG1	1:100	Sigma-Aldrich	AMAB90754
Anti-CD45	D9M8I/Rabbit IgG	1:200	Cell Signaling	13917S
Anti-Cytokeratin 7	OV-TL12/30 /Mouse IgG1	1:200	Dako	M7018
Goat anti-Mouse IgG Alexa Fluor 488	Not applicable/Goat polyclonal IgG	2 µg/ml	ThermoFisher Scientific	A-11017
Goat anti-Rabbit IgG Alexa Fluor 488	Not applicable/Goat polyclonal IgG	2 µg/ml	ThermoFisher Scientific	A-11070
Goat anti-Mouse IgG Alexa Fluor 568	Not applicable/Goat polyclonal IgG	2 µg/ml	ThermoFisher Scientific	A-11019
Goat anti-Rabbit IgG Alexa Fluor 647	Not applicable/Goat polyclonal IgG	2 µg/ml	ThermoFisher Scientific	A-21245
Anti-CD235a PE	HI264/Mouse IgG2a	2 µg/ml	BioLegend	349105
Anti-CD31 PE	REA1312/Human IgG1	1:100	Miltenyi	130-128-769
CoraLite®594 conjugated CD235a	2B12G10/Mouse IgG1	1:200	Proteintech	CL594-66778
CoraLite®Plus488 CD4	240427E12/Rabbit IgG	1:200	Proteintech	CL488-83513-7
**Antibodies for murine tissues**	**Clone name/Isotype**	**Dilution**	**Vendor**	**Cat. Nr.**
Anti-CD31	MEC 13.3/Rat IgG 2a	1:100 to 1:400	BD Pharmingen	553370

NI-A41989A_ Supplementary Tables 1 and 2

<table>
<tr><td>Anti-CD309 (Flk1)</td><td>Avas12a1/Rat IgG2a, κ</td><td>1:50</td><td>ThermoFisher Scientific</td><td>14-5821-82</td></tr>
<tr><td>Anti-Ter119</td><td>TER-119/Rat IgG2b, κ</td><td>1:100</td><td>BioLegend</td><td>116202</td></tr>
<tr><td>Anti-CD45 Alexa Fluor 647</td><td>30-F11/Rat IgG2b, κ</td><td>1:100</td><td>BioLegend</td><td>103124</td></tr>
<tr><td>Anti-CD4 BV421</td><td>RM4-5/Rat IgG2a, κ</td><td>1:25</td><td>BD OptiBuild</td><td>740007</td></tr>
<tr><td>Anti-Cytokeratin</td><td>Not applicable/Rabbit polyclonal IgG</td><td>1:10</td><td>abcam</td><td>Ab9377</td></tr>
<tr><td>Anti-CD31</td><td>2H8/ Armenian Hamster monoclonal IgG</td><td>1:100</td><td>ThermoFisher Scientific</td><td>MA3105</td></tr>
<tr><td>Anti-Laminin</td><td>Not applicable/Rabbit polyclonal IgG</td><td>1:400</td><td>Sigma Aldrich</td><td>L9393-2ML</td></tr>
<tr><td>Anti-F4/80</td><td>Rat hybridoma[10]</td><td>1:3</td><td>Not applicable</td><td></td></tr>
<tr><td>Anti-Clec4F</td><td>Not applicable/Goat polyclonal IgG</td><td>1:40</td><td>R&D Systems</td><td>AF2784</td></tr>
<tr><td>Anti-EpCAM BV421</td><td>G8.8/Rat IgG2a, κ</td><td>1:50</td><td>BD Biosciences</td><td>563214</td></tr>
<tr><td>Goat anti-Rat IgG Alexa Fluor 647</td><td rowspan="4">Not applicable/Goat polyclonal IgG</td><td rowspan="3">1:100
1:750</td><td rowspan="3">ThermoFisher Scientific</td><td>A-21247</td></tr>
<tr><td>Goat anti-Rabbit IgG Alexa Fluor 647</td><td>A-21245</td></tr>
<tr><td>Goat anti-Rabbit IgG Alexa Fluor 405</td><td>A-48254</td></tr>
<tr><td>Goat anti-Armenian Hamster IgG Alexa Fluor 647</td><td>1:100</td><td>ThermoFisher Scientific</td><td>A-78967</td></tr>
</table>

NI-A41989A_ Supplementary Tables 1 and 2

Goat anti-Rat IgG Alexa Fluor 405		1:100	ThermoFisher Scientific	A-48261
Donkey anti-Rat IgG Alexa Fluor 647	Not applicable/Donkey polyclonal IgG	1:100	abcam	Ab150155
Donkey anti-Goat AF647	Not applicable/Donkey polyclonal IgG	1:200	ThermoFisher Scientific	A21447

Supplementary Table 2: Flow cytometry antibodies

**Antigen**	**Clone**	**Dilution**	**Fluorophore**	**Vendor**	**Catalogue Number**
CD3e	145-2C11	1:200	PerCP/Cy5.5	BD Biosciences	551163
CD4	RM4-5		BV421		740007
CD11b	M1/70		BV650	BioLegend	101239
			APC/Cy7	BD Biosciences	557657
CD19	1D3		PerCP/Cy5.5	BD Biosciences	551001
CD31	390		PacBlue	BioLegend	102422
CD31	390		PECy7	BD Biosciences	561410
CD41	eBioMWReg30		PECy7	Thermo Fisher Scientific	25-0411-82
CD45	30-F11		PerCP/Cy5.5	BD Biosciences	550994
CD45	30-F11		APC/Cy7		557659
CD64	X54-5/7.1.1		PerCP/Cy5.5	BioLegend	139308

CD64	X54-5/7.1.1		AF647	BD Biosciences	558539
CD71	RI7217		PE/Cy7	BioLegend	113812
CD93	AA4.1		BV650	BD Biosciences	563807
CD115	AFS98		APC	BioLegend	135510
CD117	2B8		PE/Cy7	BD Biosciences	558163
CD135 Biotin	A2F10		N/A	Thermo Fisher Scientific	13-1351-85
EpCAM	G8.8		BV421	BD Biosciences	563214
Fc Block	2.4G2	1:1000	N/A		553142
F4/80	BM8	1:200	PerCP/Cy5.5	BioLegend	123128
F4/80	BM8		PE/Cy7		123114
Ly6G	1A8		PacBlue		127612
Ly6G	1A8		PerCP/Cy5.5	BD Biosciences	560602
NK1.1	PK136		PerCP/Cy5.5		551114
Siglec H	551		PerCP/Cy5.5	BioLegend	129614
Streptavidin APCCy7	Not applicable		APC/Cy7	BD Biosciences	554063
Ter119	TER-119		APC	BioLegend	116212
