# Supplementary material for: The maternal–fetal interface is an early hematopoietic niche enriched in distinct erythro-myeloid progenitors
Source: Nat Immunol. 2026 Oct 26;27(11):2306–21. doi: 10.1038/s41590-026-02683-4 (PMC13710174; doi:10.1038/s41590-026-02683-4)
Supplement: Supplementary file 2 [file 41590_2026_2683_MOESM2_ESM.pdf]

nature portfolio

Corresponding author(s): Prof. W. Weninger, Ap.Prof S. Tikoo and Ap. Prof. R. Jain

Last updated by author(s): 27/08/26

Reporting Summary

Nature Portfolio wishes to improve the reproducibility of the work that we publish. This form provides structure for consistency and transparency in reporting. For further information on Nature Portfolio policies, see our Editorial Policies and the Editorial Policy Checklist.

Statistics

For all statistical analyses, confirm that the following items are present in the figure legend, table legend, main text, or Methods section.

n/a	Confirmed	
☐	☒	The exact sample size (n) for each experimental group/condition, given as a discrete number and unit of measurement
☐	☒	A statement on whether measurements were taken from distinct samples or whether the same sample was measured repeatedly
☐	☒	The statistical test(s) used AND whether they are one- or two-sided<br>*Only common tests should be described solely by name; describe more complex techniques in the Methods section.*
☒	☐	A description of all covariates tested
☒	☐	A description of any assumptions or corrections, such as tests of normality and adjustment for multiple comparisons
☐	☒	A full description of the statistical parameters including central tendency (e.g. means) or other basic estimates (e.g. regression coefficient) AND variation (e.g. standard deviation) or associated estimates of uncertainty (e.g. confidence intervals)
☐	☒	For null hypothesis testing, the test statistic (e.g. F, t, r) with confidence intervals, effect sizes, degrees of freedom and P value noted<br>*Give P values as exact values whenever suitable.*
☒	☐	For Bayesian analysis, information on the choice of priors and Markov chain Monte Carlo settings
☒	☐	For hierarchical and complex designs, identification of the appropriate level for tests and full reporting of outcomes
☒	☐	Estimates of effect sizes (e.g. Cohen's d, Pearson's r), indicating how they were calculated

Our web collection on statistics for biologists contains articles on many of the points above.

Software and code

Policy information about availability of computer code

Data collection	- Flow cytometry: FACS Diva<br>- Confocal microscope(Olympus Fluoview FV3000): Olympus FV31S-SW<br>- Spinning disc confocal (Olympus IXplore SpinSR): cellSens Dimensions 4.3<br>- Confocal microscope (Leica SP8 FALCON): LAS X 3.5.7.23225<br>- Confocal microscope (Leica SP5): LASX<br>- Stereo Microscope (Zeiss Lumar.V12 Stereoscope): ZEN 2012 (blue edition)<br>- Olympus BX50 microscope:Olympus cellSens Standard software
Data analysis	Flowjo9 and 10, ImageJ, GraphPad Prism V9 to 11

For manuscripts utilizing custom algorithms or software that are central to the research but not yet described in published literature, software must be made available to editors and reviewers. We strongly encourage code deposition in a community repository (e.g. GitHub). See the Nature Portfolio guidelines for submitting code & software for further information.

Data

Policy information about availability of data

All manuscripts must include a data availability statement. This statement should provide the following information, where applicable:

- Accession codes, unique identifiers, or web links for publicly available datasets
- A description of any restrictions on data availability
- For clinical datasets or third party data, please ensure that the statement adheres to our policy

Imaging datasets are available on FigShare

Research involving human participants, their data, or biological material

Policy information about studies with human participants or human data. See also policy information about sex, gender (identity/presentation), and sexual orientation and race, ethnicity and racism.

Reporting on sex and gender	6 individual participants. All participants were female.
Reporting on race, ethnicity, or other socially relevant groupings	Not relevant
Population characteristics	Not Applicable
Recruitment	Voluntary elective non-medical pregnancy termination
Ethics oversight	The collection and use of human tissues were approved by the Independent Ethics Committee, Medical University of Vienna (see methods)

Note that full information on the approval of the study protocol must also be provided in the manuscript.

Field-specific reporting

Please select the one below that is the best fit for your research. If you are not sure, read the appropriate sections before making your selection.

☒ Life sciences ☐ Behavioural & social sciences ☐ Ecological, evolutionary & environmental sciences

For a reference copy of the document with all sections, see nature.com/documents/nr-reporting-summary-flat.pdf

Life sciences study design

All studies must disclose on these points even when the disclosure is negative.

Sample size	No statistical methods were used to predetermine sample size. The sample size was similar to previous publications. The specific sample sizes for each experiment have been detailed in their respective figure legends.
Data exclusions	No animals or individual data points were excluded. Experiments not meeting treatment conditions, predefined developmental-stage criteria or not yielding interpretable experimental data were not included.
Replication	For all figures, multiple independent experiments were conducted. All experiments were consistently reproducible.
Randomization	No prospective randomization was performed. Where possible mice were assigned randomly for timed matings.
Blinding	The researchers were not blind to the experimental conditions.

Reporting for specific materials, systems and methods

We require information from authors about some types of materials, experimental systems and methods used in many studies. Here, indicate whether each material, system or method listed is relevant to your study. If you are not sure if a list item applies to your research, read the appropriate section before selecting a response.

Materials & experimental systems

n/a	Involved in the study
	☒ Antibodies
	☒ Eukaryotic cell lines
☒	☐ Palaeontology and archaeology
	☒ Animals and other organisms
☒	☐ Clinical data
☒	☐ Dual use research of concern
☒	☐ Plants

Methods

n/a	Involved in the study
☒	☐ ChIP-seq
	☒ Flow cytometry
☒	☐ MRI-based neuroimaging

Antibodies

Antibodies used

All antibodies used in the study have been detailed in the Methods section for human antibodies and Supplementary tables 1 and 2 for murine imaging and flow experiments.

Validation

All antibodies used are commercial clones that have been employed extensively within the literature.
Pilot test experiments were performed to ensure that the staining pattern observed reflects the previously published staining patterns for various cell populations.
Other cell populations (previously described in literature) also provided an internal control for the staining patterns.
Antibodies were titrated before experimentation.

Antibodies for murine tissue staining:

Details provided as- Antibody/ Clone name/Isotype/ Dilution/ Vendor/ Cat. Nr./ Link

Anti-CD31/ MEC 13.3/ Rat IgG 2a/ 1:100 to 1:400/ BD Pharmingen/ 553370/ https://www.bdbiosciences.com/en-eu/products/reagents/microscopy-imaging-reagents/immunohistochemistry-reagents/purified-rat-anti-mouse-cd31.553370?tab=product_details

Anti-CD309 (Flk1)/ Avas12a1/ Rat IgG2a, κ/ 01:50/ ThermoFisher Scientific/ 14-5821-82/ https://www.thermofisher.com/antibody/product/CD309-FLK1-Antibody-clone-Avas12a1-Monoclonal/14-5821-82

Anti-Ter119/ TER-119/ Rat IgG2b, κ/ 1:100/ BioLegend/ 116202/ https://www.biolegend.com/en-dk/products/purified-anti-mouse-ter-119-erythroid-cells-antibody-1869?GroupID=ImportedGROUP1

Anti-CD45 Alexa Fluor 647/ 30-F11/ Rat IgG2b, κ/ 1:100/ BioLegend/ 103124/ https://www.biolegend.com/en-dk/products/alexa-fluor-647-anti-mouse-cd45-antibody-3101?GroupID=BLG6841

Anti-CD4 BV421/ RM4-5/ Rat IgG2a, κ/ 01:25/ BD OptiBuild/ 740007/ https://www.bdbiosciences.com/en-fi/products/reagents/flow-cytometry-reagents/research-reagents/single-color-antibodies-ruo/bv421-rat-anti-mouse-cd4.740007?tab=product_details

Anti-Cytokeratin/ Not applicable/ Rabbit polyclonal IgG/ 01:10 /Abcam /Ab9377/ https://www.abcam.com/en-us/products/primary-antibodies/wide-spectrum-cytokeratin-antibody-ab9377

Anti-CD31/ 2H8/ Armenian Hamster monoclonal IgG/ 1:100/ ThermoFisher Scientific/ MA3105/ https://www.thermofisher.com/antibody/product/CD31-Antibody-clone-2H8-Monoclonal/MA3105

Anti-Laminin/ Not applicable/ Rabbit polyclonal IgG/ 1:400/ Sigma Aldrich/ L9393-2ML/ https://www.sigmaaldrich.com/AT/de/product/sigma/l9393?srsltid=AfmBOorRWhZZEyMAXsGIIPa25ZiFSMhgSJR3A1HHhWRpw386FzyXhkBP

Anti-F4/80 Rat hybridoma/1:3. F4/80 antibody was from a hybridoma, Austyn et. al. 1981 (DOI: 10.1002/eji.1830111013).
Hybridoma supernatants were produced in large volume, each batch was tested on multiple tissue sections for efficient labeling of macrophages with minimal background, aliquoted and frozen at -20 degree C for future use.

Anti-Clec4F/Not applicable/Goat polyclonal IgG/1:40/R&D Systems/AF2784/https://www.rndsystems.com/products/mouse-clec4f-clecsf13-antibody_af2784?gclsrc=aw.ds&gad_source=1&gad_campaignid=20161432922&gbraid=0AAAAAD_kmX1KFr88FrHi5zJsPHNrRnpS9&gclid=CjwKCAjwwL_UBhAjEiwAEhuT5CyH0qKhdoa67fVPCfkMg_ytG-3a5z6UMUXKNNtix7mGtQ6lj0UXRBoCFA0QAvD_BwE

EpCAM BV421/G8.8/Rat IgG2a,κ/1:50/BD Biosciences/563214/https://www.bdbiosciences.com/en-at/products/reagents/flow-cytometry-reagents/research-reagents/single-color-antibodies-ruo/bv421-rat-anti-mouse-cd326.563214?tab=product_details

Donkey anti-Goat IgG Alexa Fluor 647/Not applicable/Donkey polyclonal IgG/1:200/ThermoFisher Scientific/A21447/https://www.thermofisher.com/antibody/product/Donkey-anti-Goat-IgG-H-L-Cross-Adsorbed-Secondary-Antibody-Polyclonal/A-21447

Goat anti-Rat IgG Alexa Fluor 647/ Not applicable /Goat polyclonal Ig G/ 1:100 - 1:750 /ThermoFisher Scientific/ A-21247/ https://www.thermofisher.com/antibody/product/Goat-anti-Rat-IgG-H-L-Cross-Adsorbed-Secondary-Antibody-Polyclonal/A-21247

Goat anti-Rabbit IgG Alexa Fluor 647/ Not applicable/Goat polyclonal Ig G/ 1:100 - 1:750/ThermoFisher Scientific/ A-21245 https://www.thermofisher.com/antibody/product/Goat-anti-Rabbit-IgG-H-L-Highly-Cross-Adsorbed-Secondary-Antibody-Polyclonal/A-21245

Goat anti-Rabbit IgG Alexa Fluor 405/ Not applicable/Goat polyclonal Ig G/ 1:100 - 1:750/ThermoFisher Scientific/A-48254/ https://www.thermofisher.com/antibody/product/Goat-anti-Rabbit-IgG-H-L-Highly-Cross-Adsorbed-Secondary-Antibody-Polyclonal/A48254

Goat anti-Armenian Hamster IgG Alexa Fluor 647/ Not applicable/Goat polyclonal Ig G/ 1:100/ ThermoFisher Scientific/ A-78967/ https://www.thermofisher.com/antibody/product/Goat-anti-Armenian-Hamster-IgG-H-L-Highly-Cross-Adsorbed-Secondary-Antibody-Polyclonal/A78967

Goat anti-Rat IgG Alexa Fluor 405/ Not applicable/Goat polyclonal Ig G/ 1:100/ ThermoFisher Scientific/A-48261/ https://www.thermofisher.com/antibody/product/Goat-anti-Rat-IgG-H-L-Highly-Cross-Adsorbed-Secondary-Antibody-Polyclonal/A48261

Donkey anti-Rat IgG Alexa Fluor 647/ Not applicable /Donkey polyclonal Ig G/ 1:100 /Abcam/ Ab150155/ https://www.abcam.com/en-us/products/secondary-antibodies/donkey-rat-igg-h-l-alexa-fluor-647-preadsorbed-ab150155

Antibodies for human tissue staining:
Details provided as- Antibody/ Clone name/Isotype/ Dilution/ Cat. Nr./ Vendor/Link

Anti-CD14 / D7A2T / Rabbit IgG / 1:500 / 75181S / Cell Signaling / https://www.cellsignal.com/products/primary-antibodies/cd14-d7a2t-rabbit-monoclonal-antibody-ihc-formulated/75181?srsltid=AfmBOooZJRNOMe-y3Q2oQJhHpV_foTIWF_9XnMBnIcTwzCQwwVXzdgc5

Anti-CD4 / CL0395 / Mouse IgG1 / 1:100 / AMAB90754 / Sigma / https://www.sigmaaldrich.com/AT/de/product/sigma/amab90754?srsltid=AfmBOoqMZnX27j3u0ssI1B7ZQrTBo4XlAK3GeCtoNqDVzjsk56JGaixl

Anti-CD45 / D9M8I / Rabbit IgG / 1:200 / 13917S / Cell Signaling / https://www.cellsignal.com/products/primary-antibodies/cd45-intracellular-domain-d9m8i-rabbit-monoclonal-antibody/13917?srsltid=AfmBOopYfoiSXCsga5EyQo8Wdegd3ASIxtagEkUQpiYf7W-bKKimpuI0

Anti-Cytokeratin 7 / OV-TL 12/30 / Mouse IgG1 / 1:200 / M7018 / Dako / https://www.agilent.com/en/product/immunohistochemistry/antibodies-controls/primary-antibodies/cytokeratin-7-(concentrate)-76620?srsltid=AfmBOoqE-4gH9dhg4F2nIa4h7740KpmnzuBXVuMjnpX_t5fXmYedx2V5

Goat anti-Mouse IgG Alexa Fluor 488 / Not applicable / Goat polyclonal Ig G / 2 µg/ml / A-11017 / Thermo Fisher Scientific / https://www.thermofisher.com/antibody/product/Goat-anti-Mouse-IgG-H-L-Cross-Adsorbed-Secondary-Antibody-Polyclonal/A-11017

Goat anti-Rabbit IgG Alexa Fluor 488 / Not applicable / Goat polyclonal Ig G / 2 µg/ml / A-11070 / Thermo Fisher Scientific / https://www.thermofisher.com/antibody/product/Goat-anti-Rabbit-IgG-H-L-Cross-Adsorbed-Secondary-Antibody-Polyclonal/A-11070

Goat anti-Mouse IgG Alexa Fluor 568 / Not applicable / Goat polyclonal Ig G / 2 µg/ml / A-11019 / Thermo Fisher Scientific / https://www.thermofisher.com/antibody/product/Goat-anti-Mouse-IgG-H-L-Cross-Adsorbed-Secondary-Antibody-Polyclonal/A-11019

Goat anti-Rabbit IgG Alexa Fluor 647 / Not applicable / Goat polyclonal Ig G / 2 µg/ml / A-21245 / Thermo Fisher Scientific / https://www.thermofisher.com/antibody/product/Goat-anti-Rabbit-IgG-H-L-Highly-Cross-Adsorbed-Secondary-Antibody-Polyclonal/A-21245

CD235a-PE / HI264 / Mouse IgG2a / 2 µg/ml / 349105 / BioLegend / https://www.cellsignal.com/products/primary-antibodies/cd45-intracellular-domain-d9m8i-rabbit-monoclonal-antibody/13917?srsltid=AfmBOopYfoiSXCsga5EyQo8Wdegd3ASIxtagEkUQpiYf7W-bKKimpuI0

CD31-PE / REA1312 / Human IgG1 / 1:100 / 130-128-769 / Miltenyi / https://www.miltenyibiotec.com/DE-en/products/cd31-antibody-anti-human-reafinity-rea1312.html#conjugate=pe:size=200-ul

CoraLite 594 conjugated CD235a/ Clone 2B12G10/Mouse IgG1/1:200/ThermoFisher (Proteintech)/CL594-66778/ https://www.thermofisher.com/antibody/product/Glycophorin-A-Antibody-clone-2B12G10-Monoclonal/CL59466778100UL

CoraLite Plus 488 CD4/ 240427E12/Rabbit IgG/1:200/ThermoFisher (Proteintech)/ CL488-83513-7/ https://www.thermofisher.com/antibody/product/CD4-Antibody-clone-240427E12-Recombinant-Monoclonal/CL488-83513-7

Antibodies for Flow cytometry:
Details provided as- Antibody/ Clone name/ Dilution/Fluorophore/ Vendor/ Cat. Nr./ Link

CD3e/ 145-2C11/1:200/PerCP/Cy5.5/BD Biosciences/551163/ https://www.bdbiosciences.com/en-us/products/reagents/flow-cytometry-reagents/research-reagents/single-color-antibodies-ruo/percp-cy-5-5-hamster-anti-mouse-cd3e.551163?tab=product_details

CD4/RM4-5/1:200/BV421/BD Biosciences/740007/ https://www.bdbiosciences.com/en-us/products/reagents/flow-cytometry-reagents/research-reagents/single-color-antibodies-ruo/bv421-rat-anti-mouse-cd4.740007?tab=product_details

CD11b/M1/70/1:200/BV650/BioLegend/101239/ https://www.biolegend.com/en-us/products/brilliant-violet-650-anti-mouse-human-cd11b-antibody-7638

CD11b/M1/70/1:200/APC/Cy7/BD Biosciences/557657/https://www.bdbiosciences.com/en-at/products/reagents/flow-cytometry-reagents/research-reagents/single-color-antibodies-ruo/apc-cy-7-rat-anti-cd11b.557657?tab=product_details

CD19/1D3/1:200/PerCP/Cy5.5/BD Biosciences/551001/ https://www.bdbiosciences.com/en-us/products/reagents/flow-cytometry-reagents/research-reagents/single-color-antibodies-ruo/percp-cy-5-5-rat-anti-mouse-cd19.551001?tab=product_details

CD31/390/1:200/PacBlue/BioLegend/102422/https://www.biolegend.com/en-dk/products/pacific-blue-anti-mouse-cd31-

antibody-6669

CD31/390/1:200/PECy7/BD Biosciences/561410/ https://www.bdbiosciences.com/en-us/products/reagents/flow-cytometry-reagents/research-reagents/single-color-antibodies-ruo/pe-cy-7-rat-anti-mouse-cd31.561410?tab=product_details

CD41/eBioMWReg30/1:200/PECy7/Thermo Fisher Scientific/25-0411-82/ https://www.thermofisher.com/antibody/product/CD41a-Antibody-clone-eBioMWReg30-MWReg30-Monoclonal/25-0411-82

CD45/30-F11/1:200/PerCP/Cy5.5/BD Biosciences/550994/ https://www.bdbiosciences.com/en-us/products/reagents/flow-cytometry-reagents/research-reagents/single-color-antibodies-ruo/percp-cy-5-5-rat-anti-mouse-cd45.550994?tab=product_details

CD45/30-F11/1:200/APC/Cy7/BD Biosciences/557659/ https://www.bdbiosciences.com/en-us/products/reagents/flow-cytometry-reagents/research-reagents/single-color-antibodies-ruo/apc-cy-7-rat-anti-mouse-cd45.557659?tab=product_details

CD64/X54-5/7.1.1/1:200/PerCP/Cy5.5/BioLegend/139308/ https://www.biolegend.com/en-us/products/percp-cyanine5-5-anti-mouse-cd64-fcgammari-antibody-8448

CD64/X54-5/7.1.1/1:200/AF647/BD Biosciences/558539/ https://www.bdbiosciences.com/en-us/products/reagents/flow-cytometry-reagents/research-reagents/single-color-antibodies-ruo/alexa-fluor-647-mouse-anti-mouse-cd64-a-and-b-alloantigens.558539?tab=product_details

CD71/RI7217/1:200/PE/Cy7/BioLegend/113812/ https://www.biolegend.com/en-us/products/pe-cyanine7-anti-mouse-cd71-antibody-6185

CD93/AA4.1/1:200/BV650/BDBiosciences/563807/ https://www.bdbiosciences.com/en-us/products/reagents/flow-cytometry-reagents/research-reagents/single-color-antibodies-ruo/bv650-rat-anti-mouse-cd93-early-b-lineage.740548?tab=product_details

CD115 /AFS98/1:200/APC/BioLegend/135510/ https://www.biolegend.com/en-us/products/apc-anti-mouse-cd115-csf-1r-antibody-6336

CD117 /2B8/1:200/PE/Cy7/BD Biosciences/558163/ https://www.bdbiosciences.com/en-us/products/reagents/flow-cytometry-reagents/research-reagents/single-color-antibodies-ruo/pe-cy-7-rat-anti-mouse-cd117.558163?tab=product_details

CD135 Biotin/A2F10/1:200/ Not Applicable/Thermo Fisher Scientific/13-1351-85 https://www.thermofisher.com/antibody/product/CD135-Flt3-Antibody-clone-A2F10-Monoclonal/13-1351-85

EpCAM/G8.8/1:200/BV421/BD Biosciences/563214/ https://www.bdbiosciences.com/en-us/products/reagents/flow-cytometry-reagents/research-reagents/single-color-antibodies-ruo/bv421-rat-anti-mouse-cd326.563214?tab=product_details

Fc Block/2.4G2/1:1000/ Not Applicable/BD Biosciences/553142/ https://www.bdbiosciences.com/en-us/products/reagents/western-blotting-and-molecular-reagents/purified-rat-anti-mouse-cd16-cd32-mouse-bd-fc-block.553142?tab=product_details

F4/80/BM8/1:200/PerCP/Cy5.5/BioLegend/123128/ https://www.biolegend.com/en-us/products/percp-cyanine5-5-anti-mouse-f480-antibody-4303

F4/80/BM8/1:200/PE/Cy7/BioLegend/123114/ https://www.biolegend.com/en-us/products/pe-cyanine7-anti-mouse-f4-80-antibody-4070

Ly6G/1A8/1:200/PacBlue/BioLegend/127612/ https://www.biolegend.com/en-us/products/pacific-blue-anti-mouse-ly-6g-antibody-6082

Ly6G/1A8/1:200/PerCP/Cy5.5/BD Biosciences/560602/ https://www.bdbiosciences.com/en-us/products/reagents/flow-cytometry-reagents/research-reagents/single-color-antibodies-ruo/percp-cy-5-5-rat-anti-mouse-ly-6g.560602?tab=product_details

NK1.1/PK136/1:200/PerCP/Cy5.5/BD Biosciences/551114/ https://www.bdbiosciences.com/en-us/products/reagents/flow-cytometry-reagents/research-reagents/single-color-antibodies-ruo/percp-cy-5-5-mouse-anti-mouse-nk-1-1.551114?tab=product_details

Siglec H/551/1:200/PerCP/Cy5.5/BioLegend/129614/ https://www.biolegend.com/en-us/products/percp-cyanine5-5-anti-mouse-siglec-h-antibody-6927

Streptavidin APCCy7/Not Applicable/1:200/APC/Cy7/BD Biosciences/554063/ https://www.bdbiosciences.com/en-us/products/reagents/flow-cytometry-reagents/research-reagents/single-color-antibodies-ruo/streptavidin-apc-cy-7.554063?tab=product_details

Ter119/TER-119/1:200/APC/BioLegend/116212/ https://www.biolegend.com/en-us/products/apc-anti-mouse-ter-119-erythroid-cells-antibody-1863

Eukaryotic cell lines

Policy information about cell lines and Sex and Gender in Research

Cell line source(s)	OP9 cells for differentiation were kindly provided by Prof. S. Naik, Walter and Eliza Hall Institute of Medical Research, Victoria, Australia

Authentication	All lines were authenticated by morphological and functional analysis
Mycoplasma contamination	All cell lines were tested for mycoplasma contamination and found negative in all tests.
Commonly misidentified lines (See ICLAC register)	No commonly misidentified lines were used.

Animals and other research organisms

Policy information about studies involving animals; ARRIVE guidelines recommended for reporting animal research, and Sex and Gender in Research

Laboratory animals	The details on the specific mice strains, breeding strategy, housing and age are provided in the methods section. Briefly the following strains were used:<br>-C57Bl/6J<br>-C57Bl/6JRj<br>-SWISS:Rj<br>-mT/mG<br>-DPE-GFP<br>-beta-Actin Cre<br>-CD4 Cre<br>-Ai14<br>-CD4 CreErt2
Wild animals	Not applicable
Reporting on sex	Wherever possible mice from both sexes were used. The gender of pups being analyzed was not considered in the experiments
Field-collected samples	Not applicable
Ethics oversight	All experiments were performed as per the ethics protocols approved by the animal ethics committee, Sydney Local Health District (SLHD), Animal Ethics Committee, University of Sydney (USyd), and Bundesministerium für Frauen, Wissenschaft und Forschung, Republik Österreich (Federal Ministry for Women, Science and Research, Republic of Austria). All experiments were performed using approved Standard Operating Procedures (SOPs) and flow cytometry biosafety protocols.

Note that full information on the approval of the study protocol must also be provided in the manuscript.

Plants

Seed stocks	*Report on the source of all seed stocks or other plant material used. If applicable, state the seed stock centre and catalogue number. If plant specimens were collected from the field, describe the collection location, date and sampling procedures.*
Novel plant genotypes	*Describe the methods by which all novel plant genotypes were produced. This includes those generated by transgenic approaches, gene editing, chemical/radiation-based mutagenesis and hybridization. For transgenic lines, describe the transformation method, the number of independent lines analyzed and the generation upon which experiments were performed. For gene-edited lines, describe the editor used, the endogenous sequence targeted for editing, the targeting guide RNA sequence (if applicable) and how the editor was applied.*
Authentication	*Describe any authentication procedures for each seed stock used or novel genotype generated. Describe any experiments used to assess the effect of a mutation and, where applicable, how potential secondary effects (e.g. second site T-DNA insertions, mosiacism, off-target gene editing) were examined.*

Flow Cytometry

Plots

Confirm that:

☒ The axis labels state the marker and fluorochrome used (e.g. CD4-FITC).

☒ The axis scales are clearly visible. Include numbers along axes only for bottom left plot of group (a 'group' is an analysis of identical markers).

☒ All plots are contour plots with outliers or pseudocolor plots.

☒ A numerical value for number of cells or percentage (with statistics) is provided.

Methodology

Sample preparation	Please see methods section for sample preparation for flow cytometry
Instrument	BD LSR Fortessa and BD FACS Aria II

Software	BD FACS DIVA was used to collect the data and FlowJo 9 and 10 was used for analysis
Cell population abundance	Typically more than 5000 cells were collected/ population with at least 500-1000 cells from each sorted population reanalyzed to determine sort purity. Samples with sort purity >95% were utilized for experiments.
Gating strategy	FSC/SSC were utilised for doublet exclusion to define single cells. DAPI was used for dead cell exclusion.<br>Gating strategy<br>Lineage marker cocktails were used to 'dump' labelled cells. Specific stains were then utilised to identify populations and gates were drawn based on the separation of positively and negatively stained cell populations. For further details please see Methods and Figures.

☒ Tick this box to confirm that a figure exemplifying the gating strategy is provided in the Supplementary Information.
